# Critical Analysis of Genome-Wide Association Studies: Triple Negative Breast Cancer *Quae Exempli Causa*

**DOI:** 10.3390/ijms21165835

**Published:** 2020-08-14

**Authors:** Maria-Ancuta Jurj, Mihail Buse, Alina-Andreea Zimta, Angelo Paradiso, Schuyler S. Korban, Laura-Ancuta Pop, Ioana Berindan-Neagoe

**Affiliations:** 1 Research Center for Functional Genomics, Biomedicine and Translational Medicine, Iuliu Hatieganu University of Medicine and Pharmacy, 400337 Cluj-Napoca, Cluj, Romania; ancajurj15@gmail.com (M.-A.J.); ioananeagoe29@gmail.com (I.B.-N.); 2 MEDFUTURE Research Center for Advanced Medicine, Iuliu Hatieganu University of Medicine and Pharmacy, 400337 Cluj-Napoca, Romania; da.buse@gmail.com (M.B.); zimta.alina.andreea@gmail.com (A.-A.Z.); 3 Laboratorio Oncologia Sperimentale Clinica, Istituto Oncologico-Bari, I-70124 Bari, Italy; a.paradiso@oncologico.bari.it; 4 Department of Natural Resources and Environmental Sciences, University of Illinois at Urbana-Champaign, Urbana, IL 61801, USA; korban@illinois.edu; 5 Department of Functional Genomics and Experimental Pathology, The Oncology Institute “Prof. Dr. I Chiricuta”, 400015 Cluj-Napoca, Cluj, Romania

**Keywords:** breast cancer, CNVs, GWAS, linkage disequilibrium, predictive risk scores, SNPs, structural variants, TNBC

## Abstract

Genome-wide association studies (GWAS) are useful in assessing and analyzing either differences or variations in DNA sequences across the human genome to detect genetic risk factors of diseases prevalent within a target population under study. The ultimate goal of GWAS is to predict either disease risk or disease progression by identifying genetic risk factors. These risk factors will define the biological basis of disease susceptibility for the purposes of developing innovative, preventative, and therapeutic strategies. As single nucleotide polymorphisms (SNPs) are often used in GWAS, their relevance for triple negative breast cancer (TNBC) will be assessed in this review. Furthermore, as there are different levels and patterns of linkage disequilibrium (LD) present within different human subpopulations, a plausible strategy to evaluate known SNPs associated with incidence of breast cancer in ethnically different patient cohorts will be presented and discussed. Additionally, a description of GWAS for TNBC will be presented, involving various identified SNPs correlated with miRNA sites to determine their efficacies on either prognosis or progression of TNBC in patients. Although GWAS have identified multiple common breast cancer susceptibility variants that individually would result in minor risks, it is their combined effects that would likely result in major risks. Thus, one approach to quantify synergistic effects of such common variants is to utilize polygenic risk scores. Therefore, studies utilizing predictive risk scores (PRSs) based on known breast cancer susceptibility SNPs will be evaluated. Such PRSs are potentially useful in improving stratification for screening, particularly when combining family history, other risk factors, and risk prediction models. In conclusion, although interpretation of the results from GWAS remains a challenge, the use of SNPs associated with TNBC may elucidate and better contextualize these studies.

## 1. Introduction

One of the most important discoveries of modern genetics following the release of the complete sequence of the human genome and mapping of all genes is the role of genetic variation in disease incidence. To date, there are 88 million genetic variants that have been characterized. A genetic variant is an umbrella term referring to different types of alterations in a sequence within a certain region of the genome. Although the majority of these variants are unlikely to impact human health, there are some that have been associated with disease. These associations have provided valuable information about human diseases. Such genetic variants include indels (“insertions” or “deletions”), copy number variations (CNVs), translocations, transversions, and single nucleotide polymorphisms (SNPs). For the purpose of simplicity and coherence, we will solely focus on SNPs in the present review.

SNPs are nucleotide variations occurring at a specific location within a genome, and with a frequency greater than 1% in a population. However, it should be noted that these variations do not necessarily cause disease. SNPs at low frequencies are usually referred to as mutations and, in combination with other genetic or epigenetic factors, may be responsible for causing disease. Of the aforementioned 88 million variants characterized in the human genome, 84.7 million are SNPs [1]. As SNPs have two alleles, with some exceptions wherein three alleles have been identified, there are typically two base-pair probabilities (or substitutions) for a given allele within a population [2]. SNPs are frequently used as markers for a genomic region for pursuing genetic studies, with the majority of these SNPs having minimal impacts on biological systems. For example, it has been demonstrated that, while an individual SNP or two SNPs do not appear to correlate with specific cancers, a group of 77 SNPs is found to be highly associated with breast cancer development [3]. In addition to genome-wide association studies (GWAS), the use of next-generation sequencing technologies (NGST) has also afforded the use of SNPs for both genetic mapping and evolutionary studies, as they offer unique advantages over other forms of genetic markers. Firstly, when compared with microsatellites and mitochondrial DNAs, SNPs are less affected by homoplasy, as they correspond to single base nucleotide substitutions, and thus their origins can be explained by mutation models. Secondly, in order to determine parent relatedness and population structure, SNPs have been utilized to quantify genetic variations within an individual genome. Thirdly, SNPs are highly desirable as they are present in large numbers. However, it should be noted that, as with all marker systems, there is a certain level of bias within a panel of SNPs under consideration. In order to overcome some disadvantages of the use of SNP markers in genetic studies, the use of randomly distributed SNPs throughout a genome is highly recommended for any population under investigation [4]. At a minimum, haplotype blocks within a genome should be randomly chosen [4]. Moreover, it is important to point out that SNPs and genetic mutations are neither the same, nor are they synonymous [2].

**Figure 1:**
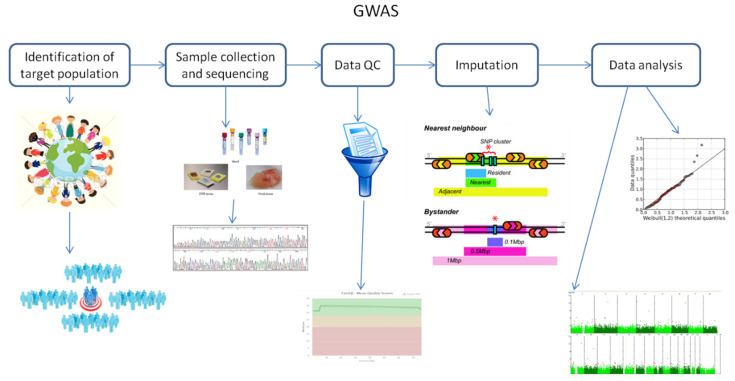
A technical flow chart for genome-wide association studies (GWAS). Abbreviation: QC, quality control.

It is known that GWAS assess and analyze differences or variations in DNA sequences across the human genome to detect genetic risk factors of diseases prevalent in a population under investigation. The ultimate goal of GWAS is to predict either disease risk or disease progression by utilizing genetic risk factors to define the biological basis of disease susceptibility. This also enables the development of innovative and preventative therapeutic strategies [2]. There are two fundamental concepts underlying GWAS, including linkage disequilibrium (LD) and a common disease–common variant (CD–CV) hypothesis. These concepts will be discussed in more detail later on in this review. A basic technical workflow for a typical GWAS is presented in Figure 1.

Therefore, GWAS serve as a tool to identify associations between genetic regions and specific traits of interest. A basic GWAS will evaluate the genetic profiles of hundreds of patients of a well-defined phenotype to those of hundreds of control subjects. In this review, we will present and discuss the most significant findings obtained thus far in GWAS for the triple negative breast cancer (TNBC) disease. We have elected to focus on the TNBC subtype mainly owing to its aggressiveness, young age of diagnosis, and limited treatment options.

## 2. Fundamental Principles of Genome-Wide Association Studies

As mentioned above, there are two fundamental concepts underlying GWAS. These are linkage disequilibrium (LD) and the common disease–common variant (CD–CV) hypothesis.

LD is defined as a non-random association of two alleles at two or more loci [5]. In turn, this provides insights into past genetic conditions and constraints, allowing for the determination of whether selection was either natural; epigenetic [6]; or owing to other mechanisms that do not occur in isolation, such as genetic drift or gene flow. If detected throughout the genome when investigating populations, LD mirrors population history, breeding system, and geographic subdivision patterns. When investigating genomic regions, LD reveals history of natural selection, gene conversion, mutation, and other forces that either contribute to or cause gene-frequency evolution. Therefore, detecting LD does not guarantee either linkage or lack of equilibrium. Ultimately, it is the local recombination rate that determines how the aforementioned factors affect LD within a certain genomic region or between paired loci [5]. As to be expected, this is related to the concept of chromosomal linkage, which infers that two markers found on a chromosome will remain physically linked on that chromosome throughout consecutive familial generations. However, recombination events will separate chromosomal segments within a family from one generation to another, and this effect is continuously amplified through several subsequent generations. Inevitably, recombination events will break apart segments of chromosomes carrying linked alleles until all alleles within a population are in linkage equilibrium. Simply stated, linkage disequilibrium involves coupling markers at the population level [2]. Furthermore, the rate of LD decay is dependent on the following factors: population size, number of founding chromosomes within a population, and number of generations for which the population has existed. Therefore, it comes as no surprise that there are different levels and patterns of LD when comparing different human subpopulations. For example, the most ancestral human population is that of an African-descent population, which, owing to the accumulation of more recombination events, has smaller regions of LD. Meanwhile, on average, European-descent and Asian-descent populations have larger regions of LD than African-descent populations. This is attributed to the fact that European- and Asian-descendant populations have been generated by founding events, whereby they have split from the African population, thus inherently changing the number of founding chromosomes, population size, and generational age of the population [2].

In general, closely linked polymorphic SNPs have strong LD between them. The International HapMap consortium has demonstrated that the human genome contains haplotype blocks, within which either most or all are high LD SNPs. Thus, there is a fine-scale pattern of LD present in human populations. Subsequently, it has been assumed that high LD levels detected among SNPs are for those alleles that exhibit increased risks of complex inherited diseases [5]. Interestingly, this has been in fact observed for those SNPs significantly associated with breast cancer when GWAS have been conducted and large numbers of SNPs have been surveyed [7]. However, it is important to take into consideration that LD in GWAS can be generated by either undetected or unknown population stratification. Moreover, GWAS have been successful at uncovering associated SNPs, despite the low overall breast cancer risk within a population, and even in identifying new causative alleles [5]. In fact, five new variants have been found to be associated with familial breast cancer, but only 3.6% of familial breast cancer can be attributed to these alleles [7]. However, it is important to point out that it is the relatively high frequency of these causative alleles that allows for their detection by GWAS.

The CD–CV hypothesis [8] has been developed based on the following two principles: common diseases differ from rare disorders in terms of their underlying genetic architecture, and the discovery of several susceptibility variants for a common disease is of high minor allele frequency [2]. In other words, this hypothesis proposes that common diseases are influenced by genetic variations common within a population [8]. Firstly, this suggests that there has to be a high correlation between allele frequency and population occurrence. Secondly, if common genetic variants influence disease, then the effect size or penetrance for any one variant must be small relative to those exhibited by rare diseases [2]. This further implies that, if the same SNP causes a small change in gene expression that alters disease risk by a small proportion, this creates a scenario wherein the frequency of disease incidence and the causal allele are only lowly-correlated. Thus, common variants cannot yield high effects. Thirdly, disease susceptibility is influenced by multiple common alleles based on the condition that common alleles have small genetic effects, and that common diseases exhibit heritability. Additionally, if an allele of a single SNP slightly increases disease risk, this implies that such an SNP accounts for a small amount of variance of the total variation caused by genetic factors. Consequently, multiple genetic factors synergistically account for the total genetic risk of a common genetic variation [2]. However, it is important not to jump to the conclusion that the entire genetic component of any disease is attributable only to common alleles.

## 3. Challenges of Genome-Wide Association Studies

While discussing genetic heterogeneity and the potential role of rare genetic variants in complex human diseases, McClellan and King [9] have pointed out some important and interesting criticisms of GWAS. Despite the fact that some of these criticisms have already been addressed, it is important to go through them to better understand these issues, and to improve the outcomes of GWAS.

One of these noted issues pertains to the fact that some of the genetic variants lack biological functions, and thereby their relative importance is highly diminished. In fact, it has been observed that GWAS are populated by risk variants of no known functions. Thus, the utility and reliability of GWAS have been questioned as most detected SNPs in GWAS are from intergenic regions [9]. Furthermore, GWAS identify approximate locations of loci associated with disease variants rather than attempt to specifically identify functional SNPs. This is attributed to widespread LD between segregating sites within a given human population. In addition, most SNPs in SNP arrays have unknown biological functions, as most SNPs in HapMaps are located in noncoding regions, and SNP arrays usually do not select for SNPs of known functions. Moreover, it is also important to emphasize that GWAS variants are not functional variants that confer risk, also referred to as “risk variants” in the published literature. Thus, 100% of a subpopulation carrying a risk allele does not truly suggest that all subjects of such a population are predisposed to risk. This simply indicates that LD patterns at a target locus are different than those of another subpopulation. Although the majority of thousands of “risk variants” that have been identified from GWAS have no apparent known biological functions, these are explained by using deduction and rationale, as outlined by the CD–CV hypothesis. This suggests that most genotyping platforms select for common variants. Moreover, as evolution has ensured that the most common variants are neutral, it should come as no surprise that most GWAS findings are neutral, originating from factors other than associations with disease risk. On the basis of evolutionary genetics, most alleles are in fact recent, and they are rare [10,11]. It is unclear what is exactly required for a common allele to remain in a population, as mechanisms of evolution can both facilitate and hinder heritability, particularly as they do not occur in isolation. For example, an allele can significantly increase in frequency without any need for selection when either a population bottleneck occurs (genetic drift) or when a subpopulation migrates and integrates with another (gene flow).

In another claim, it has been reported that it is population stratification that results in GWAS hits [9]. Although population stratification or substructure inflates test statistics, this can be readily identified, and adjusted for accordingly. In general, populations differ among each other over many loci and not only for one or two SNPs, which is precisely how whole-genome data are used to identify stratification. This is exemplified by the particularly fine-scale sub-populations in Europe that can be readily separated utilizing whole-genome data. Most importantly, as population stratification is one of the fundamental assumptions taken into consideration by the CD–CV hypothesis, the GWAS community has established methods to deal with population stratification that are fairly effective for common variants. For example, EigenStrat is a multi-dimensional scaling approach for addressing stratification, and is commonly used as a standard practice in the case-control GWAS dataset. Additionally, family-based study designs in GWAS have an advantage in protecting against stratification. Lastly, frequency estimates are dependent on sample size, thus conferring additional variations to such results [10,11].

As with all studies, sample size significantly impacts interpretations of data. Single GWAS analyses are relatively underpowered owing to the fact that they have a limited number of samples, which drastically increases the probability of false-positive findings. Given this, implementing meta-analysis of several GWAS can overcome these small-sample numbers and study-specific limitations, thus providing a more robust statistical analysis and reduced false-positive results. To date, there are many published articles describing the meta-analysis of GWAS [12,13,14]. However, each meta-analysis consists of several stages comprised of analysis set-up, investigating heterogeneity, data storage, and variant selection for any subsequent analysis. There are several parameters and methods employed for meta-analyses, such as *p*-values, fixed effects, random effects, Bayesian statistics, and multivariate analysis [15]. Using such meta-analysis methods, a new collaboration, iGOGS, has discovered 74 new susceptibly loci for hormone-dependent cancers [16,17,18,19]. However, there are other consortia that have used this method for identifying other SNPs relevant for each type of disease, such as BCAC [20], ISC [21], and MAGIC [22].

The use of GWAS for cancer research studies has encountered several challenges, including the following: sample size; high numbers (430) of significant SNPs for cancer; association of several SNPs with multiple cancer localizations; implications of identified genes in several key signaling pathways involved in cancer; modulation of some pathways by lifestyle and environment; and, lastly, the fact that most studies are conducted using European populations, thus limiting extrapolation of these findings to other populations [23].

## 4. Genome-Wide Association Studies on Triple Negative Breast Cancer

### 4.1. Triple Negative Breast Cancer

Breast cancer remains a highly serious disease with the highest female incidence and mortality, as determined by age-standardized rate (ASR) per 100,000, of 46.3 and 13.0 worldwide, 54.5 and 15.5 for Eastern Europe [24], and 51.6 and 14.6 for Romania, respectively [25]. Breast cancer is a heterogeneous disease, and it is divided into several subtypes, each with its own risk, as well as pathological and molecular characteristics. Pathologically, breast cancer is subdivided into in situ carcinoma and invasive carcinoma. The in-situ carcinoma is further subdivided into ductal and lobular, each with its own subtypes and characteristics. Meanwhile, invasive carcinoma is subdivided into tubular, ductal lobular, invasive lobular, mucinous, medullary, infiltrating ductal, and papillary [26,27]. In another classification system currently used by pathologists, several biomarkers frequently observed in breast cancer are used, including estrogen receptor (ER), progesterone receptor (PR), receptor tyrosine-protein kinase erbB-2 (HER2/neu), and p53. Expression of these biomarkers has been mainly determined for the invasive carcinoma subtype; moreover, the presence or absence of these biomarkers is correlated with responses of targeted treatments for particular patients [28]. Finally, the last breast cancer classification relies on the use of molecular profiles that separate breast cancer into the following six subtypes: basal-like [29], luminal A, luminal B, HER2-enriched, normal-like, and claudin-low [30,31,32]. Furthermore, molecular classifications of breast cancer have allowed for the use of enhanced protein staining antibodies techniques.

The above-mentioned receptor-based molecular classification undertaken by staining for estrogen, progesterone, and Her2 protein receptors has led to the identification of the breast cancer subtype triple negative breast cancer (TNBC). This subtype is the most aggressive and lethal form of all breast cancer subtypes, and is based on its elevated metastatic rate, reoccurrence, and drug resistance. Furthermore, as the nomenclature suggests, TNBC is characterized by a lack of ER, PR, and HER2/Neu expression [33,34,35]. TNBC accounts for ~10–20% of total breast cancer diagnosis, and its molecular profile reveals that it exhibits almost explicitly basal-like tumors, but not all basal-like tumors are triple negative. TNBC is pursued in numerous research studies and clinical trials because of its heterogeneity (exhibited both clinically and biologically), unfavorable outcomes, and aggressiveness. However, owing to the limited availability of targeted treatment options, conventional chemotherapy and radiotherapy are primarily still used [36,37,38,39,40]. Although there are good results obtained in many clinical trials using different targeted treatments for TNBC, there are no reported results from Phase III clinical trials, thus suggesting that the predominant treatment approach remains chemotherapy [41]. There are two Phase III clinical trials using poly (ADP-ribose) polymerase (PARP) inhibitors for treatment of breast cancer patients, yielding better results than that with chemotherapy. The first clinical trial focused on the use of Olaparib, and included patients with positive pathogenic *BRCA1/2* mutations, Her2 negative, locally advanced, or metastatic cancer that have been previously treated by chemotherapy (OlympiAD) [42]. The second clinical trial relied on the use of Talazoparib for breast cancer patients positive for *BRCA1/2* pathogenic mutations with either locally advanced or metastatic cancers (EMBRACA) [43]. In 2018 and 2019, the FDA and EU, respectively, approved the use of Olaparib for treatment of breast cancer patients with the aforementioned conditions.

### 4.2. Genome-Wide Association Studies Identifying SNPs for TNBC

Most GWAS related to cancer have evaluated either relationships of SNPs to risk of disease [23,44,45,46] or associations of SNPs to disease-specific prognosis for patients [47,48,49]. As for breast cancer, there are two studies that have associated SNPs with the risk of breast cancer and with the outcome of patients [49,50]. During the period of 2008 to 2018, a total of 41 articles were published, and retrieved in PubMed. These studies have evaluated associations of SNPs to outcomes of patients with TNBC, and of those SNPs associated with risks of developing TNBC (Table S1).

On the basis of the literature, the following genes are associated with patient prognosis: lymphocyte-specific protein 1 (*LSP1*), TOX high mobility group box family member 3 (*TOX3*), transition protein 1 (*TNP1*), carbohydrate sulfotransferase 9 (*CHST9*), aquaporin 4 (*AQP4*), BRCA1 DNA repair associated (*BRCA1*), and fibroblast growth factor receptor 2 (*FGFR2*) (Table S1). Moreover, the majority of SNPs associated with risk of breast cancer are present in the following genes: mitogen-activated protein kinase kinase kinase 1 (*MAP3K1*), RAD51 paralog B (*RAD51L1*), *BRCA1*, *BRCA2*, estrogen receptor 1 (*ESR1*), *MDM5*, transforming growth factor beta 1 (*TGFB1*), telomerase reverse transcriptase (*TERT*), carbohydrate sulfotransferase 9 (*CHST9*), or REV1 DNA directed polymerase (*REV1*). Although there is some overlap with *BRCA1/2* genes, for the most part, these associated genes are distinct. Moreover, the majority of SNPs identified in TNBC studies are associated with the risk of developing this disease, while only six SNPs are associated with either survival or prognosis of TNBC.

**Figure 2:**
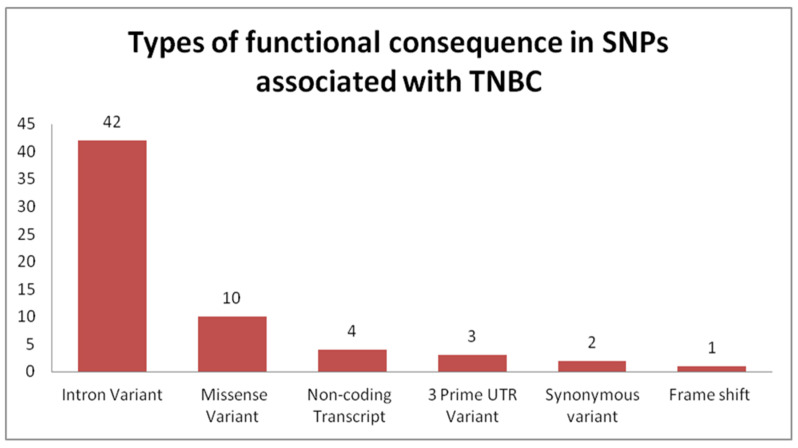
Frequencies of each type of functional consequence caused by single nucleotide polymorphisms (SNPs) associated with TNBC. Abbreviations: SNPs, single nucleotide polymorphisms; TNBC, triple negative breast cancer; UTR, untranslated region.

The most common SNPs associated with TNBC are located within introns of protein-coding genes (Figure 2 and Table S1), thus confirming that intronic variants can significantly influence the final cellular functional units. This is owing to their influence over alternative splicing [51] and generation of modified non-coding RNAs [52]. In the first instance, the SNP effect will be confined to a single protein variant, which, in turn, can either disrupt or over-activate interacting proteins belonging to various signaling pathways. Thus, knowledge of all affected interactions as a consequence of an early SNP event in breast cancer development could offer better predictions of disease prognosis and effective targeting of a central causative SNP.

In the second instance, if an intronic variant generates either long non-coding RNAs or circular RNAs [52], with either gain-of-function or loss-of-function, it will influence the expression of multiple proteins. The fallacy in this current approach of focusing on these genetic variants is that our knowledge is limited to the original protein-coding gene, but without any information on potential non-coding RNAs generated from these genetic loci. As such, for those four TNBC-associated SNPs, located in non-coding genes, there are likely several intronic variants that should also be included.

The third most common TNBC-associated SNPs are located in exons, yielding missense transcripts (Figure 2 and Table S1). This observed change directly affects protein variants, as these variants can incur either a gain-of-function or loss-of-function alteration that can, in turn, either yield an ineffective tumor suppressor or generate an oncogene. The down-stream effects of these variants are dependent on established interactions and cellular processes in which they are involved. *TGFB1* is a tumor suppressor that, upon acquisition of oncogenic mutations, begins to stimulate malignant cell invasion and metastasis through epithelial-to-mesenchymal transition [53]. The transcript variant (TGFB1):c.29C > T (p.Pro10Leu) is a risk factor for breast cancer (see Table S1). *Caspase-8* (*Casp-8*) is another interesting case, with specific effects in TNBC. In addition to its involvement in extrinsic apoptosis, *Casp-8* stimulates cell cycle progression, while it inhibits cell invasion and migration through modulation of multiple genes; thus, this Caspase may play a more profound role in TNBC than in other malignant pathologies [54]. Interestingly, for the rs1045485 SNP located within exon 10 of *Caspase 8*, it is the C allele that offers a protective role over BC development [55].

The 3′UTR variants are the fourth most common SNP-generated alterations in TNBC. These variants are most likely characterized by their altered expression of transcripts, by either creating or deleting new miRNA binding sites [56]. For instance, for the *KRAS* variant rs61764370 (A > C) containing TNBC tumors, there is a decrease in let-7 miRNA level [57]. Furthermore, synonymous variants rank fifth in terms of types of SNPs-generated variants in TNBC. Although these variants have been detected for quite a while, but without any clinical significance, more recently, they have been found to influence protein variations (through changes at splicing site preferences) and expression (creating new miRNA binding sites, less stable mRNAs, and hindering translation through changes in codon choices) [58]. *BABAM1* rs8170 (G > A) and *ALS2CR12* rs17468277 (C > T) are two synonymous genetic variants associated with an increased risk of breast cancer. Following a frameshift mutation of the *BRCA1* gene, it is detected that rs80357794 (delC) is strongly correlated with hereditary ovarian and aggressive forms of breast cancer, including TNBC (Table S1).

When analyzing the population frequency of alleles, it is important not to overlook the necessity of co-occurrence in order for a genetic variant to incur either a protective or a detrimental role. For example, *LSP1* rs3817198 has more or less the same subpopulation frequency of T and C alleles in both European and African populations; however, the C allele increases the risk of breast cancer in a European population, while it is a protective factor in an African population [59,60]. This finding is most likely owing to the incidence of associations with other SNPs in the European population, such as *BRCA1* mutations. The rs13387042 A allele homozygosity is strongly correlated with breast cancer development and aggressive development, and it is more frequently distributed in an African population. Meanwhile, the rs1436904 risk allele G is more frequently detected in American, East Asian, and European populations. Furthermore, the *SLC4A7* risk allele has a C variant that is far more common in an East Asia population in comparison with all other populations. Surprisingly, it is observed that the highest associations between this *SLC4A7* allele and incidence of ER+ breast cancer are detected in Taiwanese, Chinese, and Korean populations. Meanwhile, rs67397200 is associated with ER- breast cancer in women of European heritage. Finally, rs1219648 is the highest risk SNP for the *FGFR2* gene, and this allele has a high frequency in both European and African populations (Table S1).

**Figure 3:**
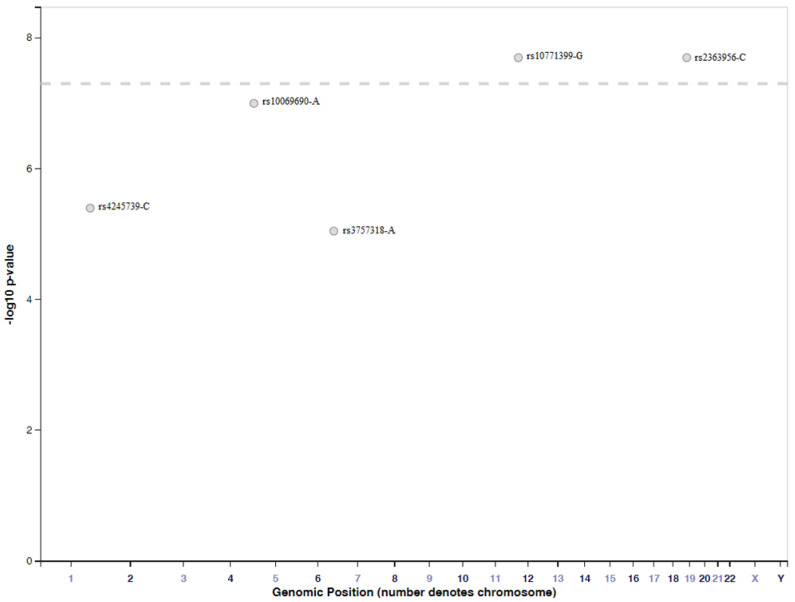
Schematic representation of SNPs associated with triple negative breast cancer obtained from the EMBL-EBI GWAS catalogue. Abbreviation: SNPs- single nucleotide polymorphism, EMBL-EBI, The European Bioinformatics Institute.

We have used the GWAS catalogue from EMBL-EBI (The European Bioinformatics Institute) to search for SNPs associated with TNBC. A total of five SNPs have been identified, and these are presented in Figure 3.

Using the GWAS catalogue from EMBL-EBI, two SNPs (rs3803662 and rs8170) were selected, based on their identification in several studies, and patterns adjacent to these two SNPs were analyzed. For rs3803662, we observed that two additional SNPs were associated with breast cancer, and a single SNP was associated with both breast cancer and Parkinson’s disease. Meanwhile, for rs8170, we identified a single SNP that was associated with ovarian cancer; three SNPs that were associated with breast cancer; and a single SNP, rs2363956, that was associated with TNBC (Figure 3). It is important to point out that the significance of identifying these few SNPs is the fact they are common in different diseases.

**Figure 4:**
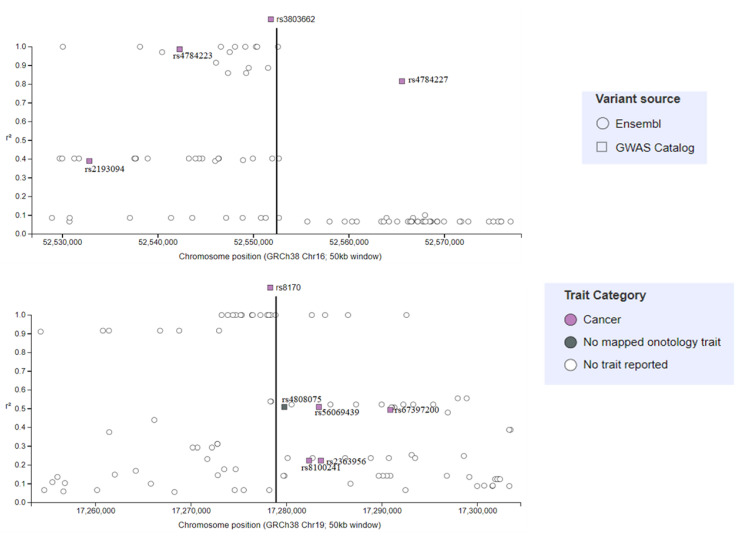
Correlations of the two most studied SNPs associated with triple negative breast cancer and other nearby SNPs identified following analysis of the GWAS catalogue. Abbreviations: SNPs, single nucleotide polymorphisms; GWAS, Genome-wide association studies.

On the basis of frequencies of different types of functional consequences generated by SNPs exhibited in TNBC, it is revealed that intronic variants are the most common functional consequence described in 42 different studies investigating SNPs in TNBC (data taken from frequencies determined from information presented in Table S1) (Figure 4).

**Figure 5:**
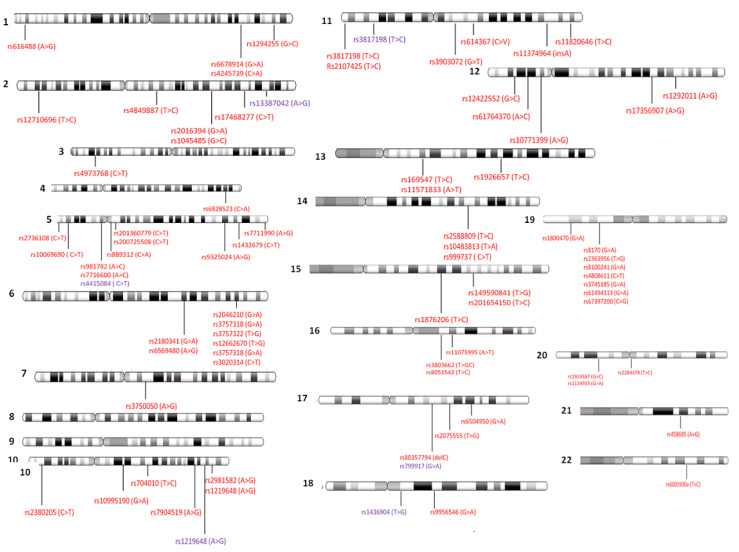
Locations of TNBC-related SNPs distributed across all somatic human chromosomes. In red color, SNPs related to TNBC risk, and in violet color, SNPs associated with different outcomes of TNBC (taken from Table S1). Abbreviations: A, adenine; C, cytosine; T, thymine; G, guanine.

Furthermore, the physical locations of each SNP associated with TNBC, previously described, along with each of the somatic chromosomes are presented in Figure 5. As can be noted, certain chromosomal locations, such as those of the q arm of chromosome 6 or the p arm of chromosome 19, are more susceptible to those SNPs detected in TNBC (Figure 5). Moreover, particular chromosomes, such as chromosome 5, have many more SNPs along various chromosomal locations when compared with other chromosomes, such as chromosomes 21 and 22 (Figure 5).

Interestingly, chromosomal locations of TNBC-related SNPs reveal that those presented in 19p13.11 and in 6q25.1-q25.2 (see Figure 5 and Table S1) are most likely in linkage disequilibrium. In 19p13.11, there are seven SNPs, including rs8170, rs2363956, rs8100241, rs4808611, rs3745185, rs61494113, and rs67397200. Meanwhile, in 6q25.1-q25.2, there are six SNPs, including rs2046210, rs3757318, rs3757322, rs12662670, rs3757318, and rs3020314.

**Figure 6:**
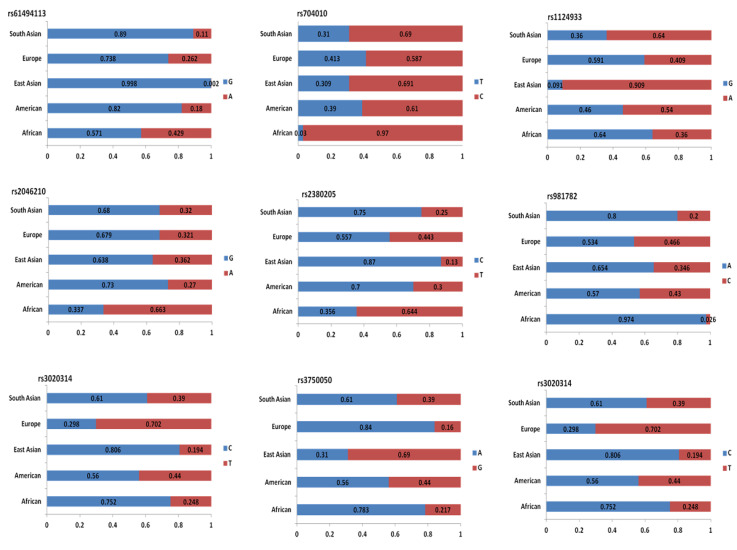
Divergent bar graphs illustrating significant differences among subpopulation frequencies noted in TNBC-related SNPs. Abbreviations: A, adenine, C, cytosine; G, guanine; T, thymine.

Among all SNPs associated with TNBC, only those SNPs with the most significant differences detected in subpopulation frequencies are presented in Figure 6. The divergent bar graphs demonstrate differences in the composition of allele frequencies exhibited within each of the five subpopulations: African, American, East-Asian, European, and South-Asian. This finding emphasizes the importance of knowledge of allele frequencies in subpopulations, especially when undertaking a GWAS.

It has been reported that approximately 14% of all TNBC cases correspond to mutations in high- and moderate-penetrance breast cancer genes [61,62]. The clinical utility of GWAS in determining those high-penetrance breast cancer gene mutations was initially very high back in the 1990s, when *BRCA1* and *BRCA2* were first cloned. Currently, it is now widely accepted that there are no other high-penetrance genes accounting for relevant proportions of familial cases. Moreover, complex diseases are now better understood; that is, individual risk for breast cancer is not determined by a single genetic variant, but rather by interactions among environmental factors, patterns in polymorphisms, and multiple genetic variants [63].

High-penetrance breast cancer genes, such as *BRCA1* and *BRCA2*, are routinely diagnosed in clinical practice in many countries. This yields a positive feedback loop, facilitating risk estimation and implementation of cancer prevention strategies [64]. Therefore, there must be a shift, in both the value and direction of clinical research, towards either moderate- or low-penetrance genes along with coupling of testing for both of these penetrance types.

New treatments and effective management strategies are in dire need for TNBC patients carrying germline mutations in genes other than *BRCA1* and *BRCA2*, as these patients account for approximately 4% of all TNBC cases [62]. Large-scale routine clinical testing of a new generation of different susceptibility alleles must be undertaken, but this will most likely come along with major technical and economic burdens [64]. Moreover, to accurately determine the overall risk of TNBC in patients, we propose that additional large-scale GWAS should be performed for ancestries other than those for European populations. A recent GWAS carried out by Mavaddat et al. [65] is the largest study to date, but its 94,075 cases and 75,017 controls are of European ancestry.

Additionally, with the advent of personalized medicine, screening programs will be adapted for a patient’s individual genetic risk. Examples of these forms of individualized patient care include decreasing age of initiation and increasing investigation intervals of mammography, as well as integration of magnetic resonance imaging and risk-reducing surgery [63]. For the highest clinical utility of these forms of screenings, we should focus on an individual’s moderate-penetrance susceptibility genes; however, the utility of such an individualized genome-based approach is yet to be validated. Currently, there appears to be little justification for complete sequencing of moderate penetrance genes, such as those of *ATM, BRIP1, CHEK2, PALB2*, and *RAD50* in *BRCA1/BRCA2*-negative high-risk breast cancer families. This is because of the fact that, in most populations, incidences of mutations in these genes are rare [63].

Therefore, despite enhanced protocols for the management and identification of patients carrying mutations for TNBC susceptibility genes, additional studies are required. Specifically, it is important to investigate and assess the clinical utility of low-penetrance genes/loci SNPs. The necessity of these additional studies has also been suggested by other researchers [65]. A highly useful tool for GWAS for investigating genetic variants is the availability of high-density SNP arrays. These arrays decode relevant variants with increased breast cancer risk in very large groups of both patients and controls. The main purpose of these arrays is to determine multiple polymorphisms that are predicted to synergistically impact an individual patient’s risk. However, it should be noted that most of these low-penetrance variants occur at such high frequencies that they can be detected in control cohorts despite their significant impact on breast cancer risk [63].

### 4.3. Importance of the Relationship between SNPs and miRNAs in BC

Triple-negative breast cancer is an aggressive form of malignancy with a high prevalence in the young female population. Although this disease has a strong genetic component, it is far less associated with environmental exposure as it is for double-positive breast cancer, lung cancer, colon cancer, cervical cancer, and others. Moreover, the majority of these mutations are germline mutations [65]. Thus far, many GWAS have generally disregarded associating these mutations with either the generation of new RNA species or the appearance of new regulatory networks as a result of these mutations. Among the wide variety of non-coding RNAs affected by SNPs are microRNAs and long non-coding RNAs (lncRNAs) [52,66,67]. We have elected to focus on microRNAs owing to the availability of a large amount of relevant data, as well as our laboratories’ experience with these RNA species.

MicroRNAs (miRNAs) are relevant to our discussion of SNPs, as they contribute to cancer risk [68]; moreover, SNPs can either create or remove miRNA binding sites [69]. Furthermore, there are limited studies on miRNA biogenesis and target selection of SNPs in miRNA genes [70]. In fact, this group of non-coding RNAs is responsible for regulating gene expression by binding to mRNA, resulting in silencing of mRNA, thereby promoting their destabilization and degradation. These miRNAs regulate gene expression of many fundamental processes within a cell, such as tumor suppression [71]. Therefore, it comes as no surprise that different SNP variants significantly alter miRNA-mediated functions [70].

Altering miRNA functions has an effect on the regulation of gene expression. Moreover, this form of polymorphism influences gene function and contributes to variability in disease susceptibility and severity in patients [72]. There is increasing evidence that altered sequences at miRNA target sites contribute to cancer [68,72,73]. For example, miR-146a and the G allele of rs2910164 have been correlated with breast cancer risk (odds ratio (OR) = 1.77; 95% confidence interval (CI) = 1.40–2.23) as its predicted binding sites are 3′ untranslated regions of *BRCA1* and *BRCA2* [74]. It is reported that there is an increased risk of ER- breast cancer (OR = 2.09; 95% CI = 1.19–3.67) in patients carrying the A allele for SNP rs743554, which is the predicted miRNA binding site for *ITGB4.* However, it should be noted that the status of HER2 is not included in this study; therefore, associations with TNBC could not be determined [72]. Furthermore, there is an increased risk of ER-breast cancer in women of African ancestry with two miRNA-associated SNPs, miR-4725 (rs73991220; OR = 1.27, 95% CI = 1.09–1.48) and *PAPD4* (rs146287903; OR = 0.49, 95% CI = 0.33–0.72) [75]. In addition, this GWAS has also found that rs72631820 in miR-339-3p is associated with increased risk in ER-positive breast cancers, while rs12355840 in miR-202 is associated with stage 1 breast cancer (OR = 0.78, *p* = 0.005) [75].

Polymorphisms in miRNAs have been linked to increased risks of several cancers, such as colon [76,77], breast [78,79], and bladder [80]. In addition, relationships of SNPs developed in miRNA networks with treatment responses and outcomes of different cancer patients have also been reported [81,82]. A tool has been developed that can correlate the effects of SNPs on miRNAs’ abilities to regulate gene expression [83]. This tool has been tested on different cancer types, including breast cancer, and it has been found that there are several miRNAs with high numbers of SNPs, such as has-mir-221-13SNPs, has-mir-141-10SNPs, and has-mir-64a-10SNPs [83]. In a control case study, it is observed that rs72631823 in pre-miRNA-34a is associated with an increased risk of triple negative breast cancer [84]. In earlier studies with three GWAS of ER-negative breast cancer patients, including the TNBC group [85,86], it has been observed that rs4245739 in the 3′UTR region of *MDM4*, creating a novel site for miR-191 [87], is specific to triple negative breast cancer patients [16]. This is further confirmed by yet another study wherein it is reported that another SNP rs34091, also located on the 3′UTR of *MDM4*, is also associated with an increased risk of triple negative breast cancer [88]. Accordingly, MirSNP [89], a single polymorphism, can affect the predicted miRNA site for either one or several miRNAs within a specific gene. All this information is summarized in Table 1.

Using the same GWAS concepts as in the case of general identification of SNPs, we can direct the observed miRNA-associated mutation frequencies towards four molecular consequences.

Firstly, modifications in miRNA promoter/enhancer regions can influence specific miRNA expression. As these forms of mutations have only been recently investigated, data are scarce. For instance, in lung cancer, the G > C SNP in −617 site and the A > G SNP in −604 site in the promoter region of miR-7 decrease the levels of expression of this miRNA, and lead to a worse prognosis of the disease [90].

Secondly, mutations in the primary miRNA processing proteins influence their function, and thus their processed miRNA availability. This was exemplified in a study of stage I and stage II breast cancer patients of African ancestry. Incidence of a higher frequency of the A allele in rs78393591, belonging to the Drosha gene, resulted in a higher risk for developing breast cancer [75].

Thirdly, mutations in miRNA sequences, especially in the miRNA seed region, would affect its binding preferences. For instance, a higher frequency of the C allele in the rs72631820 locus of miR-339-3p was associated with a higher risk of having ER-tumors in patients of African ancestry with stage I or stage II BC [75]. Meanwhile, the rs2910164 C > G in miR146a shows an increased risk of developing BC in the Australian population [74].

Lastly, mutations in the 3′UTR of one or a few miRNA targets would indirectly influence miRNAs targeting profiles of a diseased cell. For instance, women of GA or AA genotypes at the rs743554 locus have higher probabilities of developing ER-breast cancer and worse survival rates. Such a mutation in the 3′UTR of integrin β4 mRNA results in loss of tumor suppressor miRNA; that is, miR-34a ability to bind to *ITGB4* [72]. It is proposed that this causes an enhanced integrin β4 to promote tumor cell growth, survival, and invasion. This finding is supported by the observed poor survival of carriers of the variant allele. This is further supported by the fact that the miR-34 family, a direct transcriptional p53 target, down-regulates cell cycle progression genes [71]. One such example of an SNP within a gene that generates a binding site for new miRNAs is the SNP rs4245739 (C minor allele) in the 3′-UTR of the *MDM4* gene, which provides a binding site for miR-191 and miR-877-3p. It should be noted that this gain in miRNA binding site is primarily reported for small cell lung cancer and prostate cancer [69]. Despite several examples that have been provided by Moszynska et al. [70], excluding those for breast cancer, there is indeed an overlap, as the SNP rs4245739 is found to be correlated with increased risk of breast cancer development [69,88,91]. Moreover, *MDM4* is an oncoprotein that negatively regulates the p53 tumor suppressor protein, and its overexpression can lead to cancer progression [92].

In recent years, miRNAs, small non-coding RNAs that can regulate gene expression, have garnered interest owing to their capacity to act as biomarkers for both diagnosis and prognosis of diseases [93]. Interestingly, the effects of SNPs in target miRNAs sites and their interactions with disease have been extensively studied. SNPs can cause modifications in amino acids, changes in mRNA transcript stabilization, and shifts to the binding affinities of transcription factors [94]. Subsequently, several resources covering the effects of SNPs on miRNA regulation of different genes have been developed, such as Patrocles [95], dbSMR [96], MirSNP [89], and PolymiRTS [97]. In addition to all of the above mentioned examples of studies specifically focusing on miRNA binding sites, processing genes, or miRNA mutations, there is also an abundance of sequencing data that most likely contain information about miRNA-related mutations and their associations with various forms of cancer, as well as specifically with receptor negative breast cancer.

Future studies should be more focused on mutations found in intronic regions, as these might be the origins of non-coding RNA species other than those of major protein-coding mRNA. This would have a major impact on how we interpret findings from future GWAS, particularly when considering that a single miRNA mutation has a major downstream effect owing to the ability of miRNAs to interact with hundreds of mRNAs at a time.

## 5. Risk Factor Scores for Prediction of Breast Cancer

The association of disease with particular alleles remains unclear, as it should. With the availability of large numbers of published GWAS identifying disease susceptibility loci, the following studies are specifically related to breast cancer [7,98,99,100,101,102,103,104,105]. Such susceptibility loci infer the relative increased risk of a disease at a given locus, but albeit not necessarily the particular locus involved in the expression of the disease itself. This hypothesis has contributed to an interesting research paradox. If we are to assume that the disease locus is necessary for disease expression, there is little to no evidence to support such a finding. Conversely, if we are to detect an association between a disease and an allele, there is little supportive evidence beyond such an association. Unfortunately, the association itself is not sufficient for any clinical value. For these associations to be useful to a clinician or a patient, they must be correlated to predictive analytical models. One such predictive model is the predictive risk score. In this model, it is proposed that the more factors, such as environment, that are taken into consideration and accounted for by the predictive risk scores, the better the prediction and the higher the clinical value.

The following section will discuss predictive models used in some GWAS related to breast cancer, although not specifically related to TNBC, but still correlated to receptors of specific subtypes.

In the general population, it is only a small proportion of breast cancer patients who actually exhibit rare mutations in certain genes, such as *BRCA1* and *BRCA2* genes, that confer the highest risks of developing breast cancer. GWAS have discovered multiple common breast cancer susceptibility variants, which individually present small risks, but their combined effects can be substantial. One method to quantify the combined effects of common variants is to use polygenic risk scores, as these genomic profiles permit stratification of women based on their risks of developing breast cancer [106]. Currently, GWAS have identified 170 breast cancer susceptibility loci [107,108]. According to genome-wide heritability estimates, these loci account for only 40% of the heritability exhibited by all common variants on a genome-wide SNP array. This indirectly suggests that discrimination by the predictive risk score (PRS) could be improved by including more variants, and thereby widening the significance threshold. Additionally, these variants must differ based on breast cancer subtypes; for example, estrogen receptor (ER)-positive versus ER-negative. Therefore, these subtype-specific PRSs may allow for improved prediction of the disease, as well as allow for the selection of women for preventative treatments, which would be extremely beneficial for more aggressive breast cancer subtypes, such as TNBC.

Mavaddat et al. [3] developed PRSs based on 77 established breast cancer susceptibility SNPs in a study of 33,673 breast cancer cases combined with 33,381 control women of European origin. They found that women in the highest 1% of the PRS had a threefold increased risk of developing breast cancer compared with women in the middle quintile (OR = 3.36, 95% CI = 2.95–3.83). In addition, they reported that ORs for ER-positive and ER-negative disease were 3.73 (95% CI = 3.24–4.30) and 2.80 (95% CI = 2.26–3.46), respectively. Moreover, they found that lifetime risk of breast cancer for women in the lowest and highest quintiles of the PRS was 5.2% and 16.6%, respectively, for women without family history, and 8.6% and 24.4%, respectively, for women with a first-degree family history of breast cancer.

These findings highlight the potential for combining PRS and other known risk factors for risk stratification. Moreover, the risk strata defined by PRS have allowed for the evaluation of risk reduction strategies. There are various other studies that have used similar approaches by combining PRS with environmental, modifiable, or non-modifiable risk factors. For example, Garcia-Closas et al. [16] have used breast cancer as a representative model to demonstrate that genetic information, when combined with other factors, provides layered levels of risk stratification that could facilitate personal decision-making or population-based prevention programs. The breast cancer model is uniquely advantageous because it already has established modifiable risk factors, such as menopausal hormone therapy, options for chemoprevention (e.g., endocrine therapy prevents estrogen receptor positive breast cancer), and screening strategies for early detection. As mentioned above, methods for detecting low-frequency causative alleles are required. Maas et al. [109] have attempted to address this by evaluating combined risk stratification utility of common low-penetrant (small effect size) SNPs and epidemiological risk factors. A total of 17,171 cases and 19,862 controls sampled from the Breast and Prostate Cancer Cohort Consortium (BPC3), along with 5879 women participating in a 2010 National Health Interview Survey, have been taken into consideration and studied. This model is used to map the distribution of an absolute risk for the population of Caucasian women in the United States after adjusting for competing causes of mortality. The degree of stratification of absolute risk can be attributed to the following: non-modifiable factors, including SNPs, family history, and components of menstrual and reproductive history; along with modifiable factors, including body mass index (BMI), menopausal hormone therapy, alcohol consumption, and smoking. This suggests that this model permits the identification of population subsets with increased risks that would benefit from altering modifiable factors using risk-reduction strategies. One of the interesting discoveries is that women in the highest decile of risk are attributed to non-modifiable factors; that is, those women with low BMI, who do not smoke or drink, and who do not use menopausal hormone therapy (MHT) appear to have risks comparable to those of average women in the general population [109].

Rudolph et al. [107] specifically investigated how combining PRS with environmental risk factors would improve risk prediction; however, integrating PRS into risk prediction models entailed the evaluation of their joint associations with known environmental factors. Specifically, joint associations of those described by Mavaddat et al. [3], consisting of 77 SNP PRS with factors of reproductive history, alcohol consumption, menopausal hormone therapy (MHT), height, and BMI, were evaluated. These analyses were based on datasets from 20 studies consisting of up to 23,104 invasive breast cancer cases along with similar numbers of controls. Both global and tail-based goodness-of-fit tests in logistic regression models were performed with outcomes expressed for overall breast cancer, as well as for ER status. The best non-multiplicative joint associations with 77-SNP PRS were obtained for alcohol consumption (*p*-interaction = 0.009), adult height (*p*-interaction = 0.025), and the combined MHT (*p*-interaction = 0.038) specific to an ER-positive status [107].

More recently, Mavaddat et al. [65] have enhanced their earlier investigations, wherein they have generated PRS from the largest accessible genome-wide association datasets, optimized for predicting the ER-specific disease, to empirically validate PRS in prospective studies. It is reported that PRS is capable of improving stratification, specifically for screening for ER-specific PRS. Therefore, it could inform treating physicians to preventively target the use of endocrine therapies. Clinical translational studies within the framework of present screening protocols are required to assess the risks and benefits of including PRS.

## 6. Conclusions

Even though GWAS are not disease-specific, they provide important and new knowledge for both research and clinical studies. GWAS catalogues or databases offer valuable SNP libraries that assist in gaining a better understanding of a target human disease in correlation with other diseases, and also facilitate in developing new research strategies that could aid clinicians. Although most GWAS focus on European populations, these methodologies could easily be transferred to studies of other population subtypes.

As for TNBC patients, there are large numbers of identified SNPs that correlate with cancer risk, but very few SNPs that correlate with either survival or prognosis. In addition, developing a better understanding of how these alterations relate to gene and protein expression in TNBC will help in translating these findings in specific tests that could aid clinicians either in screening patients at risk for TNBC or in identifying new drugs that could improve patient outcomes by using personalized medicine.

The use of high throughput technologies would bring significant advantages to GWAS and databases, as these would correlate SNPs with other biological relevant data, and subsequently translate this complex information to patient care. Furthermore, GWAS can be used for TNBC by identifying correlations of different identified SNPs with miRNA sites useful for targeting to determine their influence on either prognosis or progression of TNBC in patients. Additionally, a functional consequence of miRNA-associated polymorphisms is variability in disease susceptibility. Furthermore, PRS can potentially improve stratification for screening, especially when combining family history, other risk factors, and risk prediction models.

In conclusion, interpretations of GWAS findings remain challenging; however, identifying and using SNPs specific for TNBC, we may be able to elucidate and better contextualize these types of studies. However, further studies should be undertaken on various other population subgroups, as well as pursuing analysis of general or healthy populations in order to overcome the limitations of our current knowledge.

## Figures and Tables

**Table 1:** Single nucleotide polymorphisms (SNPs) associated with predicted miRNA target site.

SNP	miRNA	Targeted Gene	Effect
rs4245739	hsa-miR-191-5p	*MDM4*	Create
	hsa-miR-3545-3p		Break
	hsa-miR-3669		Create
	hsa-miR-4427		Break
	hsa-miR-887		Create
rs72993667	hsa-let-7a-3p	*ESR1*	Break
	hsa-let-7b-3p		Break
	hsa-let-7f-1-3p		Break
	hsa-miR-3613-3p		Break
	hsa-miR-548n		Decrease
rs4973768	hsa-miR-302a-5p	*SLC4A7*	Create
rs4808616	hsa-miR-3121-3p	*ABHD8*	Break
	hsa-miR-3189-3p		Decrease
	hsa-miR-635		Decrease
rs61764370	hsa-miR-1262	*KRAS*	Create
	hsa-miR-34b-3p		Create
	hsa-miR-4701-3p		Create
	hsa-miR-4701-3p		Decrease

## Supplementary Materials

The following are available online at https://www.mdpi.com/1422-0067/21/16/5835/s1. Table S1: SNPs associated with triple negative breast cancer risk, prognosis, and survival, including subpopulation frequencies.
