# Supplementary material for: Critical Analysis of Genome-Wide Association Studies: Triple Negative Breast Cancer Quae Exempli Causa
Source: Int J Mol Sci. 2020 Aug 14;21(16):5835. doi: 10.3390/ijms21165835 (PMC7461549; doi:10.3390/ijms21165835)
Supplement: Supplementary file 1 [file ijms-21-05835-s001.pdf]

Supplementary

Table S1: SNPs associated with triple negative breast cancer risk, prognosis, and survival including subpopulation frequencies

SNPs	Locus	Genes	Functional consequence	Subpopulation frequency			Effects	Reference(s)
rs3817198 (T>C)	11p15	*LSP1*	Intron variant	African	T=0.863	C=0.137*; C=0.159**	Correlated with overall survival and progression-free survival and has a prognostic effect particularly in the subgroup of patients with triple-negative BC.	[1,2]
				American	T=0.82	C=0.18*; C=0.21**		
				Ashkenazi Jewish	T=0.54**	C=0.46**		
				East Asian	T=0.897*; T=0.907**	C=0.103*; C=0.093**		
				Europe	T=0.686*; T=0.7052**	C=0.314*; C=0.2948**		
				South Asian	T=0.64*	C=0.36*		
rs13387042 (A>G)	2q35	*TNP1/IGFBP5/IGFBP2/TNS1*	Non-coding transcript variant; Intron variant	African	A=0.778*; A=0.738**	G=0.222*; G=0.262**	Associated with prognostic in TNBC	[2]; [3–5]
				American	A=0.36*; A=0.38**	G=0.64*; G=0.62**		
				Ashkenazi Jewish	A=0.54**	G=0.46**		
				East Asian	A=0.097*; A=0.087**	G=0.903*; G=0.913**		
				Europe	A=0.520*; A=0.5233**	G=0.480*; G=0.4767**		
				South Asian	A=0.48*	G=0.52*		
rs1436904 (T>G)	18q11.2	*CHST9*	Intron variant	African	T=0.747*; T=0.760**	G=0.253*; G=0.240**	Novel susceptibility SNPs in TNBC. Increased risk of disease progression. Independent prognostic genetic variant in Chinese TNBC patients. Potential prognostic biomarker for early-stage of TNBC.	[6]
				American	T=0.55*; T=0.55**	G=0.45*; G=0.45**		
				Ashkenazi Jewish	T=0.66**	G=0.34**		
				East Asian	T=0.511*; T=0.503**	G=0.489*; G=0.497**		
				Europe	T=0.588*; T=0.6179**	G=0.412*; G=0.3821**		
				South Asian	T=0.76*	G=0.24*		
rs1219648	10q26	*FGFR2*	Intron variant	African	A=0.562*;	G=0.438*;	Associated with TNBC, strong	[7-9]

(A>G)					A=0.579**	G=0.421**	predictor.	
				American	A=0.59*; A=0.61**	G=0.41*; G=0.39**		
				Ashkenazi Jewish	A=0.61**	G=0.39**		
				East Asian	A=0.618*; A=0.616**	G=0.382*; G=0.384**		
				Europe	A=0.569*; A=0.6083**	G=0.431*; G=0.3917**		
				South Asian	A=0.62*	G=0.38*		
rs4415084 (C>T)	5p12	N/A	N/A	African	C=0.350*; C=0.360**	T=0.650*; T=0.640**	Associated with a worse outcome in triple negative EBC patients	[10]
				American	C=0.44*; C=0.49**	T=0.56*; T=0.51**		
				Ashkenazi Jewish	C=0.53**	T=0.47**		
				East Asian	C=0.453*; C=0.432**	T=0.547*; T=0.568**		
				Europe	C=0.591*; C=0.5904**	T=0.409*; T=0.4096**		
				South Asian	C=0.52*	T=0.48*		
rs799917 (G>A)	17q21.31	*BRCA1*	Missense variant	African	G=0.115*; G=0.1807***	A=0.885*; A=0.8193***	Might be a potential prognostic biomarker for TNBC, especially after radiotherapy.	[11,12]
				American	G=0.57*; G=0.6565***	A=0.43*; A=0.3435***		
				Asian	G=0.5232***	A=0.4768***		
				East Asian	G=0.629*	A=0.371*		
				Europe	G=0.637*; G=0.6604***	A=0.363*; A=0.3396***		
				South Asian	G=0.47*	A=0.53*		
rs889312 (C>A)	5q11	*MAP3K1/MGC 33648/MIER3*	N/A	African	C=0.337	A=0.663	Breast cancer development, slight predisposition of ER- breast cancer development. Increased susceptibility especially when associated with BRACA	[5,13–15]
				American	C=0.41	A=0.59		
				East Asian	C=0.535	A=0.465		
				Europe	C=0.283	A=0.717		
				South Asian	C=0.39	A=0.61		
rs17468277 (C>T)	2q33.1	*ALS2CR12/CA S8*	Synonymous variant	African	C=0.957	T=0.043	Slight increase in breast cancer risk. Low penetrance genetic	[5,14,16]
				American	C=0.93	T=0.07		

				Europe	C=0.880	T=0.120	variant	
				East Asian	C=0.999	T=0.001		
				South Asian	C=0.98	T=0.02		
rs999737 (C>T)	14q24.1	*RAD51L1*	Intron variant	African	C=0.992	T=0.008	Increased breast cancer risk	[17–20]
				American	C=0.83	T=0.17		
				East Asian	C=0.997	T=0.003		
				Europe	C=0.790	T=0.210		
				South Asian	C=0.91	T=0.09		
rs10069690 (C>T)	5p15	*TERT, CLPTM1L*	Intron variant	African	C=0.338	T=0.662	Increased breast cancer risk, especially in African American women	[16]; [21–24]
				American	C=0.78	T=0.22		
				East Asian	C=0.831	T=0.169		
				Europe	C=0.724	T=0.276		
				South Asian	C=0.73	T=0.27		
rs12662670 (T>G)	6q25.1	*ESR1*	Intron variant	African	T=0.961	G=0.039	Increased risk of breast cancer	[16,17,19]
				American	T=0.94	G=0.06		
				East Asian	T=0.694	G=0.306		
				Europe	T=0.919	G=0.081		
				South Asian	T=0.95	G=0.05		
rs10483813 (T>A / T>C)	14q24.1	*RAD51L1*	Intron variant	African	T=0.962	A=0.038	Increased risk of breast cancer	[16] [18]
				American	T=0.83	A=0.17		
				East Asian	T=0.976	A=0.024		
				Europe	T=0.789	A=0.211		
				South Asian	T=0.88	A=0.12		
rs8100241 (G>A)	19p13.1	*ANKLE1*	Missense variant	African	G=0.613	A=0.387	Increased risk of triple negative breast cancer development	[19]; [17,25]; [16,26,27]
				American	G=0.62	A=0.38		
				Europe	G=0.428	A=0.572		
				East Asian	G=0.688	A=0.312		
				South Asian	G=0.51	A=0.49		
rs2284378 (T>C)	20q11	*RALY/EIF2S2*	Intron variant	African	T=0.133	C=0.867	Increased susceptibility of breast cancer	[16]; [28]
				American	T=0.35	C=0.65		
				East Asian	T=0.167	C=0.833		
				Europe	T=0.292	C=0.708		
				South Asian	T=0.14	C=0.86		
rs4245739 (C>A)	1q32.1	*MDM4*	Non-coding transcript	African	C=0.231	A=0.769	Association with ER- breast cancer, increased cancer	[16,29,30]
				American	C=0.29	A=0.71		

			variant	Europe	C=0.260	A=0.740	aggressiveness when is simultaneous with P53 (Arg72Pro variant)	
				East Asian	C=0.050	A=0.950		
				South Asian	C=0.26	A=0.74		
rs11075995 (A>T)	16q12.2	*FTO*	Intron variant	African	A=0.158	T=0.842	Increased basal-like breast cancer development in Chinese women	[4,16,31]
				American	A=0.31	T=0.69		
				East Asian	A=0.322	T=0.678		
				Europe	A=0.217	T=0.783		
				South Asian	A=0.20	T=0.80		
rs1800470 (G>A / G>C)	19q13.2	*TGFB1*	Missense variant	African	G=0.414	A=0.586	Low penetrance genetic variant, commonly attributed with familial breast cancer	[16,19] [5,32]
				American	G=0.51	A=0.49		
				East Asian	G=0.555	A=0.445		
				Europe	G=0.382	A=0.618		
				South Asian	G=0.45	A=0.55		
rs4973768 (C>T)	3p24.2	*SLC4A7, NEK10*	Non-coding transcript variant	African	C=0.683	T=0.317	Low penetrance genetic variant, commonly attributed with ER+ breast cancer, especially in East Asian population	[5,19,33,34]
				American	C=0.47	T=0.53		
				East Asian	C=0.815	T=0.185		
				Europe	C=0.519	T=0.481		
				South Asian	C=0.56	T=0.44		
rs61494113 (G>A)	19p13.11	between *ANKLE1* and *A BHD8*	N/A	African	G=0.571	A=0.429	Associated with ER- breast cancer	[35]
				American	G=0.82	A=0.18		
				East Asian	G=0.998	A=0.002		
				Europe	G=0.738	A=0.262		
				South Asian	G=0.89	A=0.11		
rs67397200 (C>G)	19p13.11	N/A	N/A	African	C=0.725	G=0.275	Associated with ER- breast cancer especially in European population	[24,35,36]
				American	C=0.84	G=0.16		
				East Asian	C=0.998	G=0.002		
				Europe	C=0.738	G=0.262		
				South Asian	C=0.89	G=0.11		
rs201360779 (C>T)	5q11.2–12.1	*PDE4D*	Missense variant	African	C=1.000	T=0.000	Increased risk of TNBC development	[26]
				American	C=1.00	T=0.00		
				Europe	C=0.998	T=0.002		
				East Asian	C=1.00	T=0.00		
				Ashkenazi Jewish	C=1.00	T=0.00		
				Asian	C=1.00	T=0.00		
				South Asian	C=1.00	T=0.00		
rs200725508	5q11.2–12.1	*PDE4D*	Missense	European	C=0.9997	T=0.0003	Increased risk of TNBC	[26]

(C>T)			variant	African	C=1.000	T=0.000	development	
				East Asian	C=1.000	T=0.000		
				American	C=1.00	T=0.00		
rs201654150 (T>C)	15q22.31	*FBXL22*	Missense variant	European	T=0.99993	C=0.00007	Increased risk of TNBC development	[26]
				American	T=1.000	C=0.000		
				African	T=1.000	C=0.000		
rs149590841 (T>G)	15q22.31	*FBXL22*	Missense variant; USP3-AS1: Intron variant	African	T=1.00	G=0.00	Increased risk of TNBC development	[26]
				American	T=1.00	G=0.00		
				East Asian	T=1.00	G=0.00		
				Europe	T=1.00	G=0.00		
rs1219648 (A>G)	10q26.13	*FGFR2*	Intron variant	African	A=0.562	G=0.438	High risk allele G associated with FGFR2 mutation induced BC in post-menopausal women	[7]
				American	A=0.59	G=0.41		
				East Asian	A=0.618	G=0.382		
				Europe	A=0.569	G=0.431		
				South Asian	A=0.62	G=0.38		
rs3757322 (T>G)	6q25.1	*CCDC170*	3′ UTR variant	African	T=0.499	G=0.501	Increased risk of ER- BC	[24]
				American	T=0.75	G=0.25		
				East Asian	T=0.664	G=0.336		
				Europe	T=0.702	G=0.298		
				South Asian	T=0.70	G=0.30		
rs2046210 (G>A)	6q25	*ESR1*	N/A	African	G=0.337	A=0.663	High risk T allele of BC development in Chinese, Japanese and Americans of European origin. The African American population shows no association	[4,17,19]; [16,34,37]
				American	G=0.73	A=0.27		
				East Asian	G=0.638	A=0.362		
				Europe	G=0.679	A=0.321		
				South Asian	G=0.68	A=0.32		
rs6678914 (G>A)	1q32.1	*LGR6*	Intron variant	African	G=0.686	A=0.314	Increased risk of BC development	[4]
				American	G=0.71	A=0.29		
				East Asian	G=0.731	A=0.269		
				Europe	G=0.569	A=0.431		
				South Asian	G=0.71	A=0.29		
rs3757318 (G>A)	6q25.1	*CCDC170*	Intron variant	African	G=0.977	A=0.023	Increased risk of BC development	[4]
				American	G=0.95	A=0.05		
				East Asian	G=0.742	A=0.258		
				Europe	G=0.920	A=0.080		
				South Asian	G=0.96	A=0.04		

rs3803662 (C>T) (A>G)	16q12.1	*TNRC9*	Non-coding transcript variant	African	A=0.567	G=0.433	Homozygotes of TT allele have a significant increased risk of BC development and a slight tendency towards ER- BC	[4,5,17,19]
				American	A=0.39	G=0.61		
				East Asian	A=0.619	G=0.381		
				Europe	A=0.291	G=0.709		
				South Asian	A=0.27	G=0.73		
rs11374964 (insertion)	11q22.3	*KDELC2*	3′ UTR variant	N/A				[24]
rs8170 (G>A)	19p13	*BABAM1*	Synonymous variant	African	G=0.814	A=0.186	Increased risk of TNBC development	[16,19,23,25, 27] [4,17]
				American	G=0.90	A=0.10		
				East Asian	G=0.999	A=0.001		
				Europe	G=0.837	A=0.163		
				South Asian	G=0.91	A=0.09		
rs6678914 (G>A)	1q32.1	*LGR6*	Intron variant	African	G=0.686	A=0.314	Increased risk of BC development	[4,16]
				American	G=0.71	A=0.29		
				East Asian	G=0.731	A=0.269		
				Europe	G=0.569	A=0.431		
				South Asian	G=0.71	A=0.29		
rs4245739 (C>A)	1q32.1	*MDM4*	Non-coding transcript variant	African	C=0.231	A=0.769	Increased risk of BC development	[4,24]
				American	C=0.29	A=0.71		
				East Asian	C=0.050	A=0.950		
				Europe	C=0.260	A=0.740		
				South Asian	C=0.26	A=0.74		
rs12710696 (T>C)	2p24.1	MIR4757	Non-coding transcript variant	African	T=0.576	C=0.424	Increased risk of BC development	[4,16]
				American	T=0.35	C=0.65		
				East Asian	T=0.308	C=0.692		
				Europe	T=0.342	C=0.658		
				South Asian	T=0.59	C=0.41		
rs2736108 (C>T)	5p15.33	N/A	N/A	African	C=0.923	T=0.077	Influences telomere length and increases the risk of BC development	[4]
				American	C=0.77	T=0.23		
				East Asian	C=0.694	T=0.306		
				Europe	C=0.720	T=0.280		
				South Asian	C=0.45	T=0.55		
rs3757318	6q25.1	*ESR1*	Intron variant	African	G=0.977	A=0.023	Significantly associated with	[4,38]

(G>A)				American	G=0.95	A=0.05	BC risk in Chinese population. No significant association in German Population	
				East Asian	G=0.742	A=0.258		
				Europe	G=0.920	A=0.080		
				South Asian	G=0.96	A=0.04		
rs2363956 (T>G)	19p13.1	*ANKLE1*	Missense variant	African	T=0.495	G=0.505	Increased risk of BC development	[4,25,27]
				American	T=0.61	G=0.39		
				East Asian	T=0.688	G=0.312		
				Europe	T=0.427	G=0.573		
				South Asian	T=0.51	G=0.49		
rs616488 (A>G)	1p36.22	*PEX14*	Intron variant	African	A=0.914	G=0.086	Associated with the risk of BC development	[4]
				American	A=0.50	G=0.50		
				East Asian	A=0.683	G=0.317		
				Europe	A=0.674	G=0.326		
				South Asian	A=0.76	G=0.24		
rs4849887 (T>C)	2q14.2	*SCTR*	N/A	African	T=0.301	C=0.699	Associated with the risk of BC development	[4]
				American	T=0.13	C=0.87		
				East Asian	T=0.209	C=0.791		
				Europe	T=0.102	C=0.898		
				South Asian	T=0.25	C=0.75		
rs2016394 (G>A)	2q31.1	*DLX2-AS1*	Intron variant	African	G=0.768	A=0.232	G allele is associated with risk of BC development	[4]
				American	G=0.57	A=0.43		
				East Asian	G=0.813	A=0.187		
				Europe	G=0.542	A=0.458		
				South Asian	G=0.66	A=0.34		
rs6828523 (C>A)	4q34.1	*ADAM29*	Intron variant	African	C=0.583	A=0.417	Increased risk of BC development, especially in relation to environmental factors response	[4]
				American	C=0.82	A=0.18		
				East Asian	C=0.762	A=0.238		
				Europe	C=0.892	A=0.108		
				South Asian	C=0.78	A=0.22		
rs1432679 (C>T)	5q33.3	*EBF1*	Intron variant	African	C=0.853	T=0.147	Associated with the risk of BC development	[4]
				American	C=0.61	T=0.39		
				East Asian	C=0.611	T=0.389		
				Europe	C=0.447	T=0.553		
				South Asian	C=0.34	T=0.66		
rs7904519 (A>G)	10q25.2	*TCF7L2*	Intron variant	African	A=0.140	G=0.860	Associated with the risk of BC development	[4]
				American	A=0.65	G=0.35		

				East Asian	A=0.965	G=0.035		
				Europe	A=0.505	G=0.495		
				South Asian	A=0.62	G=0.38		
rs3903072 (G>T)	11q13.1	*OVOL1*	N/A	African	G=0.887	T=0.113	It modulates tumor microenvironment. Associated with the risk of BC development	[4,39]
				American	G=0.70	T=0.30		
				East Asian	G=0.772	T=0.228		
				Europe	G=0.540	T=0.460		
				South Asian	G=0.45	T=0.55		
rs11820646 (T>C)	11q24.3	N/A	N/A	African	T=0.228	C=0.772	Associated with the risk of TNBC development	[4]
				American	T=0.46	C=0.54		
				East Asian	T=0.478	C=0.522		
				Europe	T=0.440	C=0.560		
				South Asian	T=0.28	C=0.72		
rs12422552 (G>C)	12p13.1	N/A	N/A	African	G=0.558	C=0.442	Associated with the risk of TNBC development	[4]
				American	G=0.80	C=0.20		
				East Asian	G=0.730	C=0.270		
				Europe	G=0.710	C=0.290		
				South Asian	G=0.60	C=0.40		
rs10771399 (A>G)	12p11.22	*PTHLH*	N/A	African	A=0.966	G=0.034	Associated with the risk of TNBC development	[4]
				American	A=0.93	G=0.07		
				East Asian	A=0.823	G=0.177		
				Europe	A=0.894	G=0.106		
				South Asian	A=0.86	G=0.14		
rs17356907 (A>G)	12q22	*NTN4*	N/A	African	A=0.825	G=0.175	Increased risk of ER- BC development	[4]
				American	A=0.65	G=0.35		
				East Asian	A=0.736	G=0.264		
				Europe	A=0.707	G=0.293		
				South Asian	A=0.70	G=0.30		
rs1292011 (A>G)	12q24	*MED13L*	Intron variant	African	A=0.554	G=0.446	Increased risk of BC development, especially when associated with BRCA1/2 mutations	[4]
				American	A=0.63	G=0.37		
				East Asian	A=0.766	G=0.234		
				Europe	A=0.586	G=0.414		
				South Asian	A=0.38	G=0.62		
rs11571833 (A>T)	13q13.1	*BRCA2*	Stop gained	African	A=0.999	T=0.001	Minor single risk factor of BC	[4]
				American	A=1.00	T=0.00		
				East Asian	A=1.000	T=0.000		

				Europe	A=0.989	T=0.011		
				South Asian	A=0.99	T=0.01		
rs2588809 (T>C)	14q24.1	*RAD51L1*	Intron variant	African	T=0.309	C=0.691	Increased risk of BC development	[4]
				American	T=0.17	C=0.83		
				East Asian	T=0.028	C=0.972		
				Europe	T=0.188	C=0.812		
				South Asian	T=0.17	C=0.83		
rs6001930a (T>C)	22q13	*MLK1*	Intron variant	African	T=0.851	C=0.149	Associated with the risk of TNBC development	[4]
				American	T=0.92	C=0.08		
				East Asian	T=0.760	C=0.240		
				Europe	T=0.898	C=0.102		
				South Asian	T=0.88	C=0.12		
rs80357794 (delC)	17q21.31	*BRCA1*	Frameshift	N/A				[11]
rs169547 (T>C)	13q13.1	*BRCA2*	Missense variant	African	T=0.089	C=0.911	Associated with the risk of TNBC development, especially hereditary BC	[11,40]
				American	T=0.00	C=1.00		
				East Asian	T=0.000	C=1.000		
				Europe	T=0.001	C=0.999		
				South Asian	T=0.00	C=1.00		
rs3750050 (A>G)	7q11.23	*PTPN12*	Missense variant	African	A=0.783	G=0.217	Associated with the risk of BC development	[11]
				American	A=0.56	G=0.44		
				East Asian	A=0.310	G=0.690		
				Europe	A=0.840	G=0.160		
				South Asian	A=0.61	G=0.39		
rs1924587 (G>C)	20p11.23	*FGF4*	Intron variant	African	G=0.371	C=0.629	Associated with the risk of TNBC development	[19]
				American	G=0.55	C=0.45		
				East Asian	G=0.624	C=0.376		
				Europe	G=0.583	C=0.417		
				South Asian	G=0.70	C=0.30		
rs6504950 (G>A)	17q23.2	*STXBP4*	Intron variant	African	G=0.641	A=0.359	Associated with the risk of TNBC development	[19]
				American	G=0.82	A=0.18		
				East Asian	G=0.899	A=0.101		
				Europe	G=0.731	A=0.269		
				South Asian	G=0.81	A=0.19		
rs1926657 (T>C)	13q32.1	*ABCC4*	Intron variant	African	T=0.324	C=0.676	Associated with the risk of TNBC development	[19]
				American	T=0.22	C=0.78		

				East Asian	T=0.307	C=0.693		
				Europe	T=0.185	C=0.815		
				South Asian	T=0.31	C=0.69		
rs981782 (A>C)	5p12	*HCN1*	Intron variant	African	A=0.974	C=0.026	Associated with the risk of TNBC development	[19]
				American	A=0.57	C=0.43		
				East Asian	A=0.654	C=0.346		
				Europe	A=0.534	C=0.466		
				South Asian	A=0.80	C=0.20		
rs10995190 (G>A)	10q21.2	*ZNF365*	Intron variant	African	G=0.826	A=0.174	Associated with the risk of TNBC development	[19]
				American	G=0.89	A=0.11		
				East Asian	G=0.978	A=0.022		
				Europe	G=0.849	A=0.151		
				South Asian	G=0.92	A=0.08		
rs1124933 (G>A)	20p11.23	*NOTCH2*	None	African	G=0.640	A=0.360	Associated with the risk of TNBC development	[19]
				American	G=0.46	A=0.54		
				East Asian	G=0.091	A=0.909		
				Europe	G=0.591	A=0.409		
				South Asian	G=0.36	A=0.64		
rs3817198 (T>C)	11p15.5	*LSP1/H19*	Intron variant	African	T=0.863	C=0.137	Risk factor for BC development in European population, but it has a protective role in African population	[19] [5]
				American	T=0.82	C=0.18		
				East Asian	T=0.897	C=0.103		
				Europe	T=0.686	C=0.314		
				South Asian	T=0.64	C=0.36		
rs2981582 (A>G) (C>T)	10q26.13	*FGFR2*	Intron variant	African	A=0.495	G=0.505	Development of ER+ BC tumors if the T allele is present Associated with the risk of TNBC development	[19]
				American	A=0.42	G=0.58		
				East Asian	A=0.316	G=0.684		
				Europe	A=0.420	G=0.580		
				South Asian	A=0.34	G=0.66		
rs9325024 (A>G)	5q32	*PPP2R2B*	Intron variant	African	A=0.896	G=0.104	Associated with the risk of TNBC development	[19]
				American	A=0.77	G=0.23		
				East Asian	A=0.563	G=0.437		
				Europe	A=0.897	G=0.103		
				South Asian	A=0.70	G=0.30		
rs458685 (A>G)	21q21.3	*GRIK1*	Intron variant	African	A=0.921	G=0.079	Associated with the risk of TNBC development	[19]
				American	A=0.86	G=0.14		
				East Asian	A=0.855	G=0.145		

				Europe	A=0.828	G=0.172		
				South Asian	A=0.89	G=0.11		
rs614367 (C>V)	11q13.3	*FGF3 MYEOV:CCND1*	N/A	African	C=0.873	T=0.127	Increased risk of BC development, especially associated with BRCA1/2 mutations	[19]
				American	C=0.92	T=0.08		
				East Asian	C=0.994	T=0.006		
				European	C=0.8379	T=0.1621		
rs2075555 (T>A / T>G)	17q21.33	*COL1A1*	Intron variant	African	T=0.273	G=0.727	Associated with the risk of TNBC development	[19]
				American	T=0.31	G=0.69		
				East Asian	T=0.324	G=0.676		
				Europe	T=0.136	G=0.864		
				South Asian	T=0.24	G=0.76		
rs7716600 (A>C)	5p12	*MRPS30*	N/A	African	A=0.170	C=0.830	Increased risk of TNBC development	[19]
				American	A=0.30	C=0.70		
				East Asian	A=0.482	C=0.518		
				Europe	A=0.204	C=0.796		
				South Asian	A=0.26	C=0.74		
rs9956546 (G>A)	18q12.2	*FHOD3*	Intron variant	African	G=0.630	A=0.370	Increased risk of TNBC development	[19]
				American	G=0.74	A=0.26		
				East Asian	G=0.520	A=0.480		
				Europe	G=0.886	A=0.114		
				South Asian	G=0.80	A=0.20		
rs7711990 (A>G)	5q35.3	*BTNL8*	Intron variant	African	A=0.336	G=0.664	Increased risk of TNBC development	[19]
				American	A=0.55	G=0.45		
				East Asian	A=0.654	G=0.346		
				Europe	A=0.596	G=0.404		
				South Asian	A=0.73	G=0.27		
rs2180341 (G>A)	6q22.33	*RNF146*	Intron variant	African	G=0.305	A=0.695	Increased risk of TNBC development	[19]
				American	G=0.23	A=0.77		
				East Asian	G=0.197	A=0.803		
				Europe	G=0.280	A=0.720		
				South Asian	G=0.41	A=0.59		
rs1294255 (G>C)	1q42.2	*K1AA1804 MAP3K21*	Intron variant	African	G=0.860	C=0.140	Decreased risk of lymph node metastasis	[19,41]
				American	G=0.77	C=0.23		
				East Asian	G=0.614	C=0.386		
				Europe	G=0.615	C=0.385		
				South Asian	G=0.66	C=0.34		

rs2380205 (C>T)	10p15.1	*ANKRD16: FBXO18*	N/A	African	C=0.356	T=0.644	When associated with BRCA1/2 mutation, it induces great risk of BC development	[19]
				American	C=0.70	T=0.30		
				East Asian	C=0.870	T=0.130		
				Europe	C=0.557	T=0.443		
				South Asian	C=0.75	T=0.25		
rs704010 (T>C)	10q22.3	*TERT/ZMIZ1*	Intron variant	African	T=0.030	C=0.970	When associated with BRCA1/2 mutation, it induces great risk of BC development	[19,23]
				American	T=0.39	C=0.61		
				East Asian	T=0.309	C=0.691		
				Europe	T=0.413	C=0.587		
				South Asian	T=0.31	C=0.69		
rs6569480 (A>G)	6q22.33	*ECHDC1*	Intron variant	African	A=0.306	G=0.694	Increased risk of BC development	[19]
				American	A=0.23	G=0.77		
				East Asian	A=0.197	G=0.803		
				Europe	A=0.280	G=0.720		
				South Asian	A=0.41	G=0.59		
rs1045485 (G>C)	2q33.1	*CASP8*	Missense variant	African	G=0.950	C=0.050	C allele offers protection against BC development	[19,42]
				American	G=0.92	C=0.08		
				East Asian	G=0.999	C=0.001		
				Europe	G=0.880	C=0.120		
				South Asian	G=0.98	C=0.02		
rs3020314 (C>T)	6q25.1	*ESR1*	Intron variant	African	C=0.752	T=0.248	C alleles causes a predisposition for ER+ BC tumors development	[19,43]
				American	C=0.56	T=0.44		
				East Asian	C=0.806	T=0.194		
				Europe	C=0.298	T=0.702		
				South Asian	C=0.61	T=0.39		
rs1876206 (T>C)	15q21.1	*FBN1*	Intron variant	African	T=0.939	C=0.061	Increased risk of BC development	[19]
				American	T=0.88	C=0.12		
				East Asian	T=0.872	C=0.128		
				Europe	T=0.851	C=0.149		
				South Asian	T=0.86	C=0.14		
rs8051542 (T>C)	16q12.1	*TOX3*	Intron variant	African	T=0.292	C=0.708	Increased risk of BC development	[19]
				American	T=0.43	C=0.57		
				East Asian	T=0.178	C=0.822		
				Europe	T=0.432	C=0.568		
				South Asian	T=0.28	C=0.72		
rs2107425	11p15.5	*H19*	Intron variant;	African	T=0.292	C=0.708	Increased risk of BC	[19]

(T>C)			MRPL23; : Intron variant	American	T=0.43	C=0.57	development	
				East Asian	T=0.178	C=0.822		
				Europe	T=0.432	C=0.568		
				South Asian	T=0.28	C=0.72		
rs61764370 (A>C)	12p12.1	*KRAS*	3′ UTR variant	African	A=0.994	C=0.006	Increased risk of BC development	[44]
				American	A=0.93	C=0.07		
				East Asian	A=1.000	C=0.000		
				Europe	A=0.904	C=0.096		
				South Asian	A=0.98	C=0.02		
rs4808611 (C>T)	19p13.11	*NR2F6*	Intron variant	African	C=0.844	T=0.156	Increased risk of BC development	[27]
				American	C=0.91	T=0.09		
				East Asian	C=0.999	T=0.001		
				Europe	C=0.841	T=0.159		
				South Asian	C=0.91	T=0.09		
rs3745185 (G>A)	19p13.11	*BABAM1*	Intron variant	African	G=0.794	A=0.206	Increased risk of BC development	[27]
				American	G=0.68	A=0.32		
				East Asian	G=0.851	A=0.149		
				Europe	G=0.521	A=0.479		
				South Asian	G=0.56	A=0.44		

References

1. Thomas, G.; Jacobs, K.B.; Kraft, P.; Yeager, M.; Wacholder, S.; Cox, D.G.; Hankinson, S.E.; Hutchinson, A.; Wang, Z.; Yu, K., et al. A multistage genome-wide association study in breast cancer identifies two new risk alleles at 1p11.2 and 14q24.1 (RAD51L1). *Nature genetics* **2009**, *41*, 579-584, doi:10.1038/ng.353.
2. Hein, A.; Rack, B.; Li, L.; Ekici, A.B.; Reis, A.; Lux, M.P.; Cunningham, J.M.; Rubner, M.; Fridley, B.L.; Schneeweiss, A., et al. Genetic Breast Cancer Susceptibility Variants and Prognosis in the Prospectively Randomized SUCCESS A Study. *Geburtshilfe und Frauenheilkunde* **2017**, *77*, 651-659, doi:10.1055/s-0042-113189.
3. Liang, H.; Li, H.; Yang, X.; Chen, L.; Zhu, A.; Sun, M.; Ye, C.; Li, M. Associations of Genetic Variants at Nongenic Susceptibility Loci with Breast Cancer Risk and Heterogeneity by Tumor Subtype in Southern Han Chinese Women. *BioMed research international* **2016**, *2016*, 3065493, doi:10.1155/2016/3065493.
4. Purrington, K.S.; Slager, S.; Eccles, D.; Yannoukakos, D.; Fasching, P.A.; Miron, P.; Carpenter, J.; Chang-Claude, J.; Martin, N.G.; Montgomery, G.W., et al. Genome-wide association study identifies 25 known breast cancer susceptibility loci as risk factors for triple-negative breast cancer. *Carcinogenesis* **2014**, *35*, 1012-1019, doi:10.1093/carcin/bgt404.
5. Broeks, A.; Schmidt, M.K.; Sherman, M.E.; Couch, F.J.; Hopper, J.L.; Dite, G.S.; Apicella, C.; Smith, L.D.; Hammet, F.; Southey, M.C., et al. Low penetrance breast cancer susceptibility loci are associated with specific breast tumor subtypes: findings from the Breast Cancer Association Consortium. *Human molecular genetics* **2011**, *20*, 3289-3303, doi:10.1093/hmg/ddr228.
6. Yuan, J.; Zhang, N.; Zhu, H.; Liu, J.; Xing, H.; Ma, F.; Yang, M. CHST9 rs1436904 genetic variant contributes to prognosis of triple-negative breast cancer. *Scientific reports* **2017**, *7*, 11802, doi:10.1038/s41598-017-12306-6.

7. Han, M.R.; Deming-Halverson, S.; Cai, Q.; Wen, W.; Shrubsole, M.J.; Shu, X.O.; Zheng, W.; Long, J. Evaluating 17 breast cancer susceptibility loci in the Nashville breast health study. *Breast cancer (Tokyo, Japan)* **2015**, *22*, 544-551, doi:10.1007/s12282-014-0518-2.
8. Lei, H.; Deng, C.X. Fibroblast Growth Factor Receptor 2 Signaling in Breast Cancer. *International journal of biological sciences* **2017**, *13*, 1163-1171, doi:10.7150/ijbs.20792.
9. Wang, Y.; Zhang, H.; Lin, M.; Wang, Y. Association of FGFR2 and PI3KCA genetic variants with the risk of breast cancer in a Chinese population. *Cancer management and research* **2018**, *10*, 1305-1311, doi:10.2147/cmar.s164084.
10. Fu, F.; Guo, W.; Lin, Y.; Zeng, B.; Qiu, W.; Huang, M.; Wang, C. Subtype-specific associations between breast cancer risk polymorphisms and the survival of early-stage breast cancer. *Journal of translational medicine* **2018**, *16*, 270, doi:10.1186/s12967-018-1634-0.
11. Horvath, A.; Pakala, S.B.; Mudvari, P.; Reddy, S.D.; Ohshiro, K.; Casimiro, S.; Pires, R.; Fuqua, S.A.; Toi, M.; Costa, L., et al. Novel insights into breast cancer genetic variance through RNA sequencing. *Scientific reports* **2013**, *3*, 2256, doi:10.1038/srep02256.
12. Shi, M.; Ma, F.; Liu, J.; Xing, H.; Zhu, H.; Yu, J.; Yang, M. A functional BRCA1 coding sequence genetic variant contributes to prognosis of triple-negative breast cancer, especially after radiotherapy. *Breast cancer research and treatment* **2017**, *166*, 109-116, doi:10.1007/s10549-017-4395-1.
13. Garcia-Closas, M.; Hall, P.; Nevanlinna, H.; Pooley, K.; Morrison, J.; Richesson, D.A.; Bojesen, S.E.; Nordestgaard, B.G.; Axelsson, C.K.; Arias, J.I., et al. Heterogeneity of breast cancer associations with five susceptibility loci by clinical and pathological characteristics. *PLoS genetics* **2008**, *4*, e1000054, doi:10.1371/journal.pgen.1000054.
14. Odefrey, F.; Stone, J.; Gurrin, L.C.; Byrnes, G.B.; Apicella, C.; Dite, G.S.; Cawson, J.N.; Giles, G.G.; Treloar, S.A.; English, D.R., et al. Common genetic variants associated with breast cancer and mammographic density measures that predict disease. *Cancer Res* **2010**, *70*, 1449-1458, doi:10.1158/0008-5472.Can-09-3495.
15. Fanale, D.; Amodeo, V.; Corsini, L.R.; Rizzo, S.; Bazan, V.; Russo, A. Breast cancer genome-wide association studies: there is strength in numbers. *Oncogene* **2012**, *31*, 2121-2128, doi:10.1038/onc.2011.408.
16. Stevens, K.N.; Vachon, C.M.; Couch, F.J. Genetic susceptibility to triple-negative breast cancer. *Cancer research* **2013**, *73*, 2025-2030, doi:10.1158/0008-5472.can-12-1699.
17. Stevens, K.N.; Vachon, C.M.; Lee, A.M.; Slager, S.; Lesnick, T.; Olswold, C.; Fasching, P.A.; Miron, P.; Eccles, D.; Carpenter, J.E., et al. Common breast cancer susceptibility loci are associated with triple-negative breast cancer. *Cancer research* **2011**, *71*, 6240-6249, doi:10.1158/0008-5472.can-11-1266.
18. Figueroa, J.D.; Garcia-Closas, M.; Humphreys, M.; Platte, R.; Hopper, J.L.; Southey, M.C.; Apicella, C.; Hammet, F.; Schmidt, M.K.; Broeks, A., et al. Associations of common variants at 1p11.2 and 14q24.1 (RAD51L1) with breast cancer risk and heterogeneity by tumor subtype: findings from the Breast Cancer Association Consortium. *Human molecular genetics* **2011**, *20*, 4693-4706, doi:10.1093/hmg/ddr368.
19. Hicks, C.; Kumar, R.; Pannuti, A.; Backus, K.; Brown, A.; Monico, J.; Miele, L. An Integrative Genomics Approach for Associating GWAS Information with Triple-Negative Breast Cancer. *Cancer informatics* **2013**, *12*, 1-20, doi:10.4137/cin.s10413.
20. Campa, D.; Kaaks, R.; Le Marchand, L.; Haiman, C.A.; Travis, R.C.; Berg, C.D.; Buring, J.E.; Chanock, S.J.; Diver, W.R.; Dostal, L., et al. Interactions between genetic variants and breast cancer risk factors in the breast and prostate cancer cohort consortium. *Journal of the National Cancer Institute* **2011**, *103*, 1252-1263, doi:10.1093/jnci/djr265.
21. Haiman, C.A.; Chen, G.K.; Vachon, C.M.; Canzian, F.; Dunning, A.; Millikan, R.C.; Wang, X.; Ademuyiwa, F.; Ahmed, S.; Ambrosone, C.B., et al. A common variant at the TERT-CLPTM1L locus is associated with estrogen receptor-negative breast cancer. *Nature genetics* **2011**, *43*, 1210-1214, doi:10.1038/ng.985.
22. Jiao, Q.; Wu, A.; Shao, G.; Peng, H.; Wang, M.; Ji, S.; Liu, P.; Zhang, J. The latest progress in research on triple negative breast cancer (TNBC): risk factors, possible therapeutic targets and prognostic markers. *Journal of thoracic disease* **2014**, *6*, 1329-1335, doi:10.3978/j.issn.2072-1439.2014.08.13.

23. Palmer, J.R.; Ruiz-Narvaez, E.A.; Rotimi, C.N.; Cupples, L.A.; Cozier, Y.C.; Adams-Campbell, L.L.; Rosenberg, L. Genetic susceptibility loci for subtypes of breast cancer in an African American population. *Cancer epidemiology, biomarkers & prevention : a publication of the American Association for Cancer Research, cosponsored by the American Society of Preventive Oncology* **2013**, *22*, 127-134, doi:10.1158/1055-9965.epi-12-0769.
24. Milne, R.L.; Kuchenbaecker, K.B.; Michailidou, K.; Beesley, J.; Kar, S.; Lindstrom, S.; Hui, S.; Lemacon, A.; Soucy, P.; Dennis, J., et al. Identification of ten variants associated with risk of estrogen-receptor-negative breast cancer. *Nature genetics* **2017**, *49*, 1767-1778, doi:10.1038/ng.3785.
25. Stevens, K.N.; Fredericksen, Z.; Vachon, C.M.; Wang, X.; Margolin, S.; Lindblom, A.; Nevanlinna, H.; Greco, D.; Aittomaki, K.; Blomqvist, C., et al. 19p13.1 is a triple-negative-specific breast cancer susceptibility locus. *Cancer research* **2012**, *72*, 1795-1803, doi:10.1158/0008-5472.can-11-3364.
26. Haddad, S.A.; Ruiz-Narvaez, E.A.; Haiman, C.A.; Sucheston-Campbell, L.E.; Bensen, J.T.; Zhu, Q.; Liu, S.; Yao, S.; Bandera, E.V.; Rosenberg, L., et al. An exome-wide analysis of low frequency and rare variants in relation to risk of breast cancer in African American Women: the AMBER Consortium. *Carcinogenesis* **2016**, *37*, 870-877, doi:10.1093/carcin/bgw067.
27. Antoniou, A.C.; Wang, X.; Fredericksen, Z.S.; McGuffog, L.; Tarrell, R.; Sinilnikova, O.M.; Healey, S.; Morrison, J.; Kartsonaki, C.; Lesnick, T., et al. A locus on 19p13 modifies risk of breast cancer in BRCA1 mutation carriers and is associated with hormone receptor-negative breast cancer in the general population. *Nature genetics* **2010**, *42*, 885-892, doi:10.1038/ng.669.
28. Siddiq, A.; Couch, F.J.; Chen, G.K.; Lindstrom, S.; Eccles, D.; Millikan, R.C.; Michailidou, K.; Stram, D.O.; Beckmann, L.; Rhie, S.K., et al. A meta-analysis of genome-wide association studies of breast cancer identifies two novel susceptibility loci at 6q14 and 20q11. *Human molecular genetics* **2012**, *21*, 5373-5384, doi:10.1093/hmg/dds381.
29. Garcia-Closas, M.; Couch, F.J.; Lindstrom, S.; Michailidou, K.; Schmidt, M.K.; Brook, M.N.; Orr, N.; Rhie, S.K.; Riboli, E.; Feigelson, H.S., et al. Genome-wide association studies identify four ER negative-specific breast cancer risk loci. *Nature genetics* **2013**, *45*, 392-398, 398e391-392, doi:10.1038/ng.2561.
30. Liu, J.; Tang, X.; Li, M.; Lu, C.; Shi, J.; Zhou, L.; Yuan, Q.; Yang, M. Functional MDM4 rs4245739 genetic variant, alone and in combination with P53 Arg72Pro polymorphism, contributes to breast cancer susceptibility. *Breast cancer research and treatment* **2013**, *140*, 151-157, doi:10.1007/s10549-013-2615-x.
31. Zhang, B.; Li, Y.; Li, L.; Chen, M.; Zhang, C.; Zuo, X.B.; Zhou, F.S.; Liang, B.; Zhu, J.; Li, P., et al. Association study of susceptibility loci with specific breast cancer subtypes in Chinese women. *Breast cancer research and treatment* **2014**, *146*, 503-514, doi:10.1007/s10549-014-3041-4.
32. Vitiello, G.A.F.; Guembarovski, R.L.; Hirata, B.K.B.; Amarante, M.K.; de Oliveira, C.E.C.; de Oliveira, K.B.; Cebinelli, G.C.M.; Guembarovski, A.L.; Campos, C.Z.; Watanabe, M.A.E. Transforming growth factor beta 1 (TGFbeta1) polymorphisms and haplotype structures have dual roles in breast cancer pathogenesis. *Journal of cancer research and clinical oncology* **2018**, *144*, 645-655, doi:10.1007/s00432-018-2585-9.
33. Lin, C.Y.; Ho, C.M.; Bau, D.T.; Yang, S.F.; Liu, S.H.; Lin, P.H.; Lin, T.H.; Tien, N.; Shih, M.C.; Lu, J.J. Evaluation of breast cancer susceptibility loci on 2q35, 3p24, 17q23 and FGFR2 genes in Taiwanese women with breast cancer. *Anticancer research* **2012**, *32*, 475-482.
34. Han, W.; Woo, J.H.; Yu, J.H.; Lee, M.J.; Moon, H.G.; Kang, D.; Noh, D.Y. Common genetic variants associated with breast cancer in Korean women and differential susceptibility according to intrinsic subtype. *Cancer epidemiology, biomarkers & prevention : a publication of the American Association for Cancer Research, cosponsored by the American Society of Preventive Oncology* **2011**, *20*, 793-798, doi:10.1158/1055-9965.epi-10-1282.
35. Lawrenson, K.; Kar, S.; McCue, K.; Kuchenbaeker, K.; Michailidou, K.; Tyrer, J.; Beesley, J.; Ramus, S.J.; Li, Q.; Delgado, M.K., et al. Functional mechanisms underlying pleiotropic risk alleles at the 19p13.1 breast-ovarian cancer susceptibility locus. *Nature communications* **2016**, *7*, 12675, doi:10.1038/ncomms12675.
36. Feng, Y.; Rhie, S.K.; Huo, D.; Ruiz-Narvaez, E.A.; Haddad, S.A.; Ambrosone, C.B.; John, E.M.; Bernstein, L.; Zheng, W.; Hu, J.J., et al. Characterizing Genetic Susceptibility to Breast Cancer in Women of African Ancestry. *Cancer epidemiology, biomarkers & prevention : a publication of the American Association for Cancer Research, cosponsored by the American Society of Preventive Oncology* **2017**, *26*, 1016-1026, doi:10.1158/1055-9965.EPI-16-0567.

37. Cai, Q.; Wen, W.; Qu, S.; Li, G.; Egan, K.M.; Chen, K.; Deming, S.L.; Shen, H.; Shen, C.Y.; Gammon, M.D., et al. Replication and functional genomic analyses of the breast cancer susceptibility locus at 6q25.1 generalize its importance in women of chinese, Japanese, and European ancestry. *Cancer Res* **2011**, *71*, 1344-1355, doi:10.1158/0008-5472.Can-10-2733.
38. Barzan, D.; Veldwijk, M.R.; Herskind, C.; Li, Y.; Zhang, B.; Sperk, E.; Du, W.D.; Zhang, X.J.; Wenz, F. Comparison of genetic variation of breast cancer susceptibility genes in Chinese and German populations. *European journal of human genetics : EJHG* **2013**, *21*, 1286-1292, doi:10.1038/ejhg.2013.38.
39. Zhang, Y.; Manjunath, M.; Yan, J.; Baur, B.A.; Zhang, S.; Roy, S.; Song, J.S. The Cancer-Associated Genetic Variant Rs3903072 Modulates Immune Cells in the Tumor Microenvironment. *Frontiers in genetics* **2019**, *10*, 754, doi:10.3389/fgene.2019.00754.
40. Nykamp, K.; Anderson, M.; Powers, M.; Garcia, J.; Herrera, B.; Ho, Y.Y.; Kobayashi, Y.; Patil, N.; Thusberg, J.; Westbrook, M., et al. Sherloc: a comprehensive refinement of the ACMG-AMP variant classification criteria. *Genetics in medicine : official journal of the American College of Medical Genetics* **2017**, *19*, 1105-1117, doi:10.1038/gim.2017.37.
41. Shan, J.; Chouchane, A.; Mokrab, Y.; Saad, M.; Boujassoum, S.; Sayaman, R.W.; Ziv, E.; Bouaouina, N.; Remadi, Y.; Gabbouj, S., et al. Genetic Variation in CCL5 Signaling Genes and Triple Negative Breast Cancer: Susceptibility and Prognosis Implications. *Front Oncol* **2019**, *9*, 1328, doi:10.3389/fonc.2019.01328.
42. Cox, A.; Dunning, A.M.; Garcia-Closas, M.; Balasubramanian, S.; Reed, M.W.; Pooley, K.A.; Scollen, S.; Baynes, C.; Ponder, B.A.; Chanock, S., et al. A common coding variant in CASP8 is associated with breast cancer risk. *Nat Genet* **2007**, *39*, 352-358, doi:10.1038/ng1981.
43. Dunning, A.M.; Healey, C.S.; Baynes, C.; Maia, A.T.; Scollen, S.; Vega, A.; Rodriguez, R.; Barbosa-Morais, N.L.; Ponder, B.A.; Low, Y.L., et al. Association of ESR1 gene tagging SNPs with breast cancer risk. *Human molecular genetics* **2009**, *18*, 1131-1139, doi:10.1093/hmg/ddn429.
44. Paranjape, T.; Heneghan, H.; Lindner, R.; Keane, F.K.; Hoffman, A.; Hollestelle, A.; Dorairaj, J.; Geyda, K.; Pelletier, C.; Nallur, S., et al. A 3'-untranslated region KRAS variant and triple-negative breast cancer: a case-control and genetic analysis. *The Lancet. Oncology* **2011**, *12*, 377-386, doi:10.1016/s1470-2045(11)70044-4.

© 2020 by the authors. Submitted for possible open access publication under the terms and conditions of the Creative Commons Attribution (CC BY) license (http://creativecommons.org/licenses/by/4.0/).
